# FARSB serves as a novel hypomethylated and immune cell infiltration related prognostic biomarker in hepatocellular carcinoma

**DOI:** 10.18632/aging.204619

**Published:** 2023-04-03

**Authors:** Jing Zhen, Jingying Pan, Xuanrui Zhou, Zichuan Yu, Yike Jiang, Yiyang Gong, Yongqi Ding, Yue Liu, Liangyun Guo

**Affiliations:** 1 Second Affiliated Hospital of Nanchang University, Nanchang, China; 2 Second College of Clinical Medicine, Nanchang University, Nanchang, China; 3 Department of Ultrasonography, Second Affiliated Hospital of Nanchang University, Nanchang, China

**Keywords:** FARSB, hepatocellular carcinoma, prognosis, immune infiltration, m6A

## Abstract

Purpose: Hepatocellular carcinoma (HCC) is a prevalent tumor with high morbidity, and an unfavourable prognosis. FARSB is an aminoacyl tRNA synthase, and plays a key role in protein synthesis in cells. Furthermore, previous reports have indicated that FARSB is overexpressed in gastric tumor tissues and is associated with a poor prognosis and tumorigenesis. However, the function of FARSB in HCC has not been studied.

Results: The results showed that FARSB mRNA and protein levels were upregulated in HCC and were closely related to many clinicopathological characteristics. Besides, according to multivariate Cox analysis, high FARSB expression was linked with a shorter survival time in HCC and may be an independent prognostic factor. In addition, the FARSB promoter methylation level was negatively associated with the expression of FARSB. Furthermore, enrichment analysis showed that FARSB was related to the cell cycle. And TIMER analysis revealed that the FARSB expression was closely linked to tumor purity and immune cell infiltration. The TCGA and ICGC data analysis suggested that FARSB expression is greatly related to m6A modifier related genes. Potential FARSB-related ceRNA regulatory networks were also constructed. What’s more, based on the FARSB-protein interaction network, molecular docking models of FARSB and RPLP1 were constructed. Finally, drug susceptibility testing revealed that FARSB was susceptible to 38 different drugs or small molecules.

Conclusions: FARSB can serve as a prognostic biomarker for HCC and provide clues about immune infiltration, and m6A modification.

## INTRODUCTION

Hepatocellular carcinoma (HCC) is the most common type of primary liver cancer and constitutes more than 90% of the primary tumor of the liver. HCC is now the world’s fifth most widespread cause of cancer and the second leading cause of cancer death [1]. Nowadays, the morbidity of HCC worldwide is still rising, owing to the absence of obvious signs in the early days and rapid development of HCC [2]. Moreover, the five-year rate of survival for HCC is only 18%, as more than 60% of HCC patients are identified in late stages and have a poor prognosis [3]. At present, the test of serum alpha-fetoprotein (AFP), abdominal ultrasound, and triple-phase helical CT are the main diagnostic methods for HCC [4–7]. Regrettably, the accuracy and specificity of these approaches are still unsatisfying, especially in the early stages of disease [8]. Meanwhile, numerous studies have demonstrated that biomarkers display promising diagnostic abilities in HCC, such as AFP-L3, GP73, DCP, GPC3, SCCA, and OPN [9, 10]. So far, recognition of effective biomarkers in HCC monitoring and early diagnosis remains deficient. As a result, there is a pressing need to evaluate innovative treatment targets and early diagnostic markers to enhance the prognosis of HCC patients.

FARSB (Phenylalanyl-TRNA Synthetase Subunit Beta) is an aminoacyl tRNA synthase (ARSs), it has been shown to be associated with brain calcification, interstitial lung disease and liver cirrhosis [11]. Nevertheless, liver cirrhosis is one of the leading causes of hepatocellular carcinoma. ARSs are essential enzymes for protein synthesis in cells [12]. As molecular adapters translate mRNA into proteins, they are already present in the most primitive prokaryotes [13]. Recent studies suggest that ARSs play key roles in controlling transcription, translation, splicing, apoptosis, inflammation, immune response, tumorigenesis, and other important physiological and pathological processes [14, 15], implying that ARSs have potential as novel therapeutic targets and drugs in multiple pathways [16]. It also takes a part in biological processes closely linked to the development of cancer. To exemplify, meththiamide-tRNA synthetase combines with CDK4 and promotes cell cycle, tyrosine-trna synthases can also act as secreted cytokines to shape the tumor microenvironment [17, 18]. And moreover, ARSs are participated in the pathway of tumorigenesis by combining with proteins that interact with ARS. In HCC, AIMP3, one of the macromolecular protein complex cofactors of ARSs, is reduced in HCC [19], and overexpression of glycyl-tRNA synthetase (GARS) was considered for a biomarker of poor prognosis in patients [20]. Recent report contained that FARSB could be a prognosis biomarker in gastric cancer [21]. Nevertheless, the research about FARSB biological function and mechanism in hepatocellular carcinoma has not been put forward, and FARSB correlation with prognosis is unclear.

In our study, the possible participation mechanism of FARSB in liver cancer was analyzed using bioinformatics methods based on data from multiple online open databases. To start with, the relation between differential expression of FARSB in hepatocellular carcinoma and patients’ poor prognosis was explored, and included the mechanism of FARSB in liver cancer, such as tumor infiltrating immune cells, m6a methylation and drug sensitivity. Our study is the first to find out the important function of FARSB in HCC, and demonstrates a possible mechanism of FARSB in HCC, for example, the recruitment of certain immune cells. In conclusion, our results suggest that FARSB is a novel hepatocellular carcinoma biomarker and may be used as a prognostic factor for human HCC.

## MATERIALS AND METHODS

### Data collection and processing

HCC clinical and mRNA expression level had been collected from the TCGA Database and ICGC Database. For TCGA, as far as the gene expression profile is concerned, that research included 374 LIHC samples and 50 normal samples, and the data variety of mRNA expression profile was HTSeq-FPKM. And our group obtained clinical information from 377 patients. Besides, RNA-seq data were also collected from the ICGC website (https://dcc.icgc.org/projects/LIRI-JP). [LINC-JP] Liver Cancer - NCC, JP datasets included 202 normal samples and 243 tumor samples [22]. Furthermore, we also downloaded RNA-seq data and clinical information from GEO database (https://www.ncbi.nlm.nih.gov/geo/). The GSE76421 datasets have 52 normal samples and 112 tumor samples.

### TIMER database analysis

TIMER (https://cistrome.shinyapps.io/timer/) is a library of capabilities for systematic analysis of immune infiltrations in different cancer types [23, 24] which contains 1000 samples of thirty-two different tumor types. In tumor tissue, the relationship between RNA-seq expression profile data and the immune cell infiltration degree could be embodied. In other words, it can reflect the correlation hidden in cancer and immune cells. We analyzed expression of FARSB in different tumors and interaction of FARSB expression and immune infiltration, along with conventional immune cells. After that we utilized SCAN model to find out the correlation of immune cell infiltration and FARSB CNV. Additionally, our researcher utilized TIMER to study the interaction hidden in FARSB expression and various immune gene markers. Subsequently, our team visualized the correlation of FARSB expression level and immune checkpoint gene expression level by the correlation module.

### TISCH analysis

TISCH (http://tisch.comp-genomics.org/) gives us a user-friendly tool to systematically visualize, search and download gene expression atlas of the tumor microenvironment from various cancer types [25]. We utilized TISCH to expound FARSB expression level in different immune cells.

### cBioPortal analysis

The cBioPortal for Cancer Genomics (https://www.cbioportal.org/) is a website resource for exploring, visualizing, and analyzing multidimensional cancer genomics data [26]. This website helped us study association of FARSB promoter methylation level and expression in HCC with “Plot” module [27]. Additionally, the “Mutations” module was utilized to found out secondary structure of FARSB and its physical interaction protein in HCC.

### HPA analysis

HPA (https://www.Proteinatlas.org/) uses antibody methods for immunostaining of tissues and cell lines, along with contrasting expression analysis of proteins in normal and tumor tissues. For our analysis, the immunohistochemical outcomes of FARSB in tumor tissues and normal tissues were concluded by HPA database.

### GEPIA analysis

Gene expression profiling transactional estimation GEPIA (http://gepia.cancer-pku.cn/) is an interactional website app consisting of tumor and normal tissue sample data, which can be used to visualize clinicopathological characteristics [28]. The tumor data come from the TCGA database. Furthermore, the m6A-related genes LRPPRC, RBM15B and HNRNPA2B1’s overall survival in HCC was also displayed in this website.

### HCCDB analysis

HCCDB: Integrative Molecular Database of Hepatocellular Carcinoma (http://lifeome.net/database/hccdb) is a free to use database which include 15 open access online datasets for researchers [29]. During our article, it accomplished the mission that demonstrate differential expression of FARSB in HCC successfully.

### Protein-protein interaction network (PPI) analysis

The online website (STRING, https://string-db.org) for finding interacting genes was utilized for PPI network construction and pivot gene screening [30]. Besides, with the purpose of exploring the correlation amongst the top 500, our team used STRING database for analysis, medium confidence=0.4 for screening, and visualization using Cytoscape software. To search cluster sub-networks, the Cytoscape Molecular Complex Detection (MCODE) plug-in has been utilized [31]. The acquiescent parameters are listed below: degree cutoff = 5, node score cutoff = 0.2, k-core = 9, maximum depth = 100.

### GSCALite analysis

GSCALite (http://bioinfo.life.hust.edu.cn/web/GSCALite/) is a multifunctional genomics online portal for analyzing various bioinformatics-related information [32]. In our research, the GSCALite was used to analyze the pathway activities and drug sensitivity of FARSB, FARSA and USP8.

### Kaplan-Meier plotter database analysis

The Kaplan-Meier database (http://kmplot.com/analysis/) can be utilized to appraise effect of genes on the survival of tumor tissue samples [33, 34]. We used more than three hundred LIHC samples to evaluate the interaction hidden in FARSB expression and overall survival (OS), relapse-free survival (RFS), progression-free survival (PFS), and disease specific survival (DSS). We also explored the difference in LIHC patients’ survival under different immune cell subtypes. A 95% confidence interval and logrank p < 0.05 were studied as analytically important.

### UALCAN analysis

UALCAN (http://ualcan.path.uab.edu/) is an interactive evaluation and excavation website of the Internet, which can be used to analyze the relationship between tumor and normal specimens and the relative expression of genes with diverse clinicopathological characteristics [35, 36]. UALCAN was used to investigate various clinicopathological characteristics of FARSB and the methylation of FARSB promoter. The research further utilized UALCAN to compare patients' survival time between FARSB higher expression and FARSB less expression in different clinicopathological characteristics.

### GeneMANIA analysis

GeneMANIA (genemania.org) can locate genes whose connected with input genes [37, 38]. We apply its tool to identify the gene which has physical interaction with FARSB.

### LinkedOmics database analysis

LinkedOmics database (http://www.linkedomics.org/login.php) contains multi-omics data and clinical data for 32 cancer types and a total of 11158 patients from The Cancer Genome Atlas (TCGA) project [39]. We utilized the LinkFinder module of LinkedOmics to research the differently expressed genes connected with FARSB in TCGA LIHC (n = 515). We performed statistical analysis on the conclusions and display them in volcano maps and heat maps. Gene set enrichment analysis (GSEA) has been utilized for LinkedOmics functional modules to conduct gene ontology (GO) analysis and KEGG pathway analysis. False discovery rate (FDR) less than 0.01 is a noteworthy expression, P-values less than 0.05 is a significant related gene.

### Prediction and construction of ceRNA networks

The TargetScan (http://www.targetscan.org), DIANA-microT (http://diana.imis.athena-innovation.gr/DianaTools/index) and RNAinter (http://www.rnainter.org) online websites have been used to find out target miRNAs of FARSB (the miRNA which appears in three databases at the same time and have negative correlation with FARSB will be confirmed). At next step, miRNet2.0 (www.mirnet.ca/miRNet/home.xhtml) and starBase3.0 (www.starbase.sysu.edu.cn) were put to use to forecast target lncRNAs of the screened miRNAs (the lncRNA which appears in two databases at the same time and have negative correlation with target miRNA will be confirmed). In the last stage, an lncRNA-miRNA-mRNA (FARSB) ceRNA network in HCC will be completed by researcher.

### Statistical analysis

R software (version 3.6.3/4.1.2) had been utilized to complete all statistical analyses amongst our research. The “limma”, “ggplot2” and “bee swarm” packages of “R” and the rank-sum test were utilized to detect the difference of FARSB expressions between LIHC samples and normal samples. Logistic regression was utilized to scrutinize the relevancy between FARSB expression and clinicopathological features. Afterwards, we used the Kaplan-Meier method to generate survival curves. Besides, the “survival” and “survminer” packages of “ R were used to construct Cox models, and the elements striking related to prognosis had filtered out by Univariate and Multivariate Cox regression analysis (P <0.05). After that, the ROC curve created by “survival ROC” was utilized to estimate the predictive ability of FARSB expression level to one-year, three-year, or five-year survival.

### Protein data bank database analysis

Protein data bank (PDB) (https://www.rcsb.org/) is the single worldwide archive of structural data of biological macromolecules [40, 41]. We utilized PDB to find out spatial structures of FARSB and its related proteins. The structure of FARSB comes from PDB ID: 3L4G, and RPLP1 comes from PDB ID: 2LBF. Then we through the ZDOCK (https://zdock.umassmed.edu/) to study the pattern of combined docking between FARSB and RPLP1, and visualization used PYMOL (version 1.5.0.3).

### Data availability statement

All the data used in this study are publicly available.

## RESULTS

### The FARSB expression is striking increased in HCC patients

**Figure 1:**
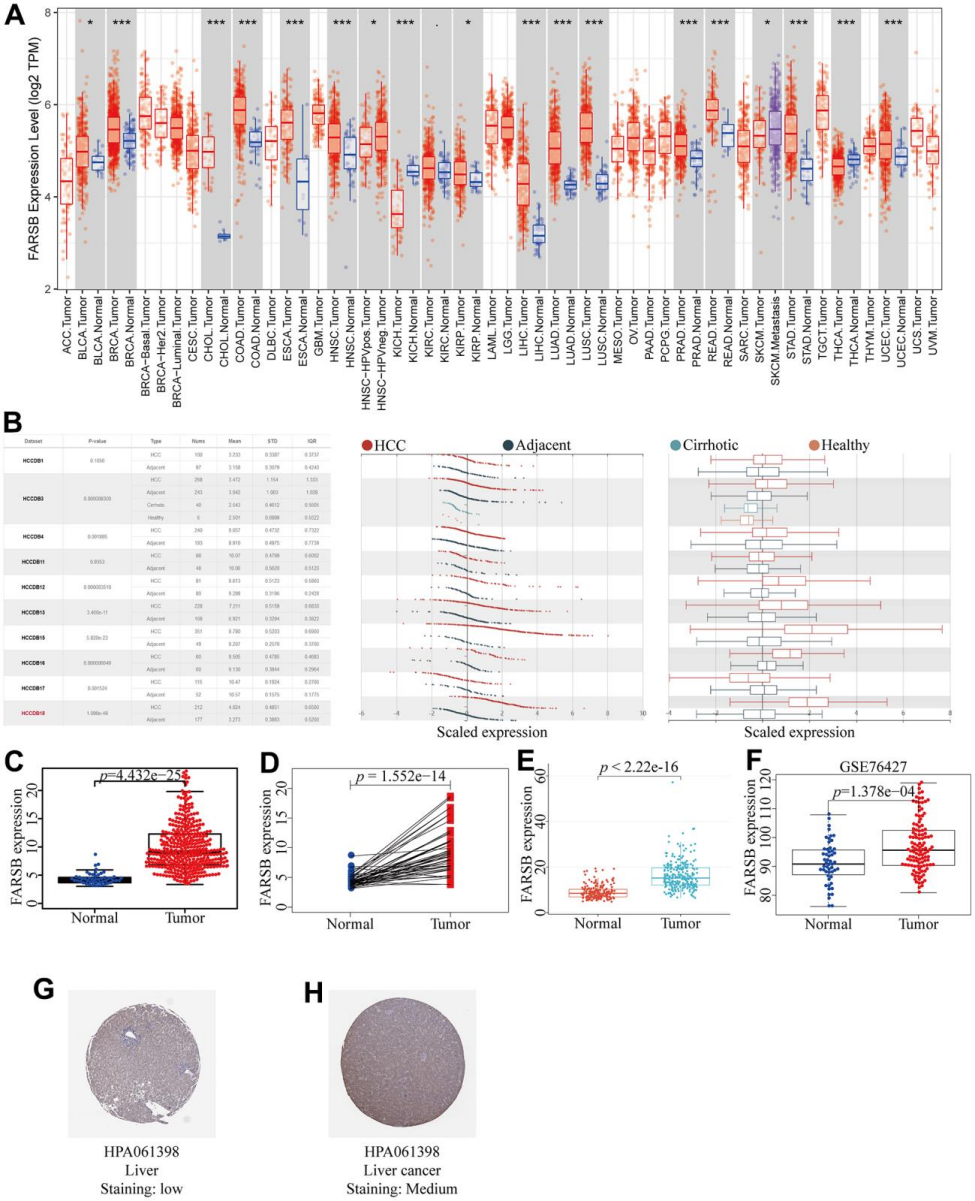
**Expression of FARSB in HCC.** (**A**) The expression level of FARSB in different types of tumor tissues and normal tissues in the TIMER database (*p* < 0.05). (**B**) Expression levels of FARSB in HCC tissues and adjacent by HCCDB datasets. (**C**) Expression levels of FARSB were higher than corresponding normal tissues in LIHC samples (TCGA-LIHC) (*p*=4.432e-25). (**D**) FARSB expression in 50 paired LIHC tissues and corresponding adjacent non-tumor tissues (TCGA-LIHC) (*p*=1.552e-14). (**E**) Expression levels of FARSB were higher than corresponding normal tissues in LIHC samples by using ICGC-LIRI-JP liver datasets (*p*<2.22e-16). (**F**) Expression levels of FARSB were higher than corresponding normal tissues in LIHC samples by using GEO GSE76427 liver cancer survival datasets (*p*=1.376e-04). (**G**, **H**) FARSB protein expression in normal and LIHC tissues (HPA).

With the aim of illuminating FARSB expression in HCC, we utilized the TCGA, TIMER, HCCDB, and ICGC databases firstly to plot different forms of visual representation. According to TIMER databases, we discovered that FARSB expression levels in HCC and many other tumors were significantly up-regulated (Figure 1A). For the sake of reliability, we also utilized the HCCDB to validate our results. The experimental results were in line with our expectation that FARSB expression in HCC tissues was higher than that in normal tissues (Figure 1B). Next, we compared 50 adjacent normal tissue samples and 377 HCC samples, and the result indicated that FARSB expression was clearly increased (Figure 1C). After that, contrast with adjacent normal samples, FARSB expression in 58 pairs of LIHC tumor samples was significantly increased (Figure 1D). Meanwhile, the result of Figure 1C was also verified by data sets LIRI-JP (liver cancer-RIKEN, JP) downloaded from the ICGC and GSE76427 downloaded from GEO (Figure 1E, 1F). Additionally, based on HPA data, our team confirmed that the Phenylalanine--tRNA ligase beta subunit expression level of tumor tissues in the liver of patients with HCC was remarkably higher than normal tissues. (Figure 1G, 1H). In brief, FARSB expression was higher in HCC tissues than in normal tissues.

### Clinicopathologic features of HCC patients could be affected by FARSB expression

**Figure 2:**
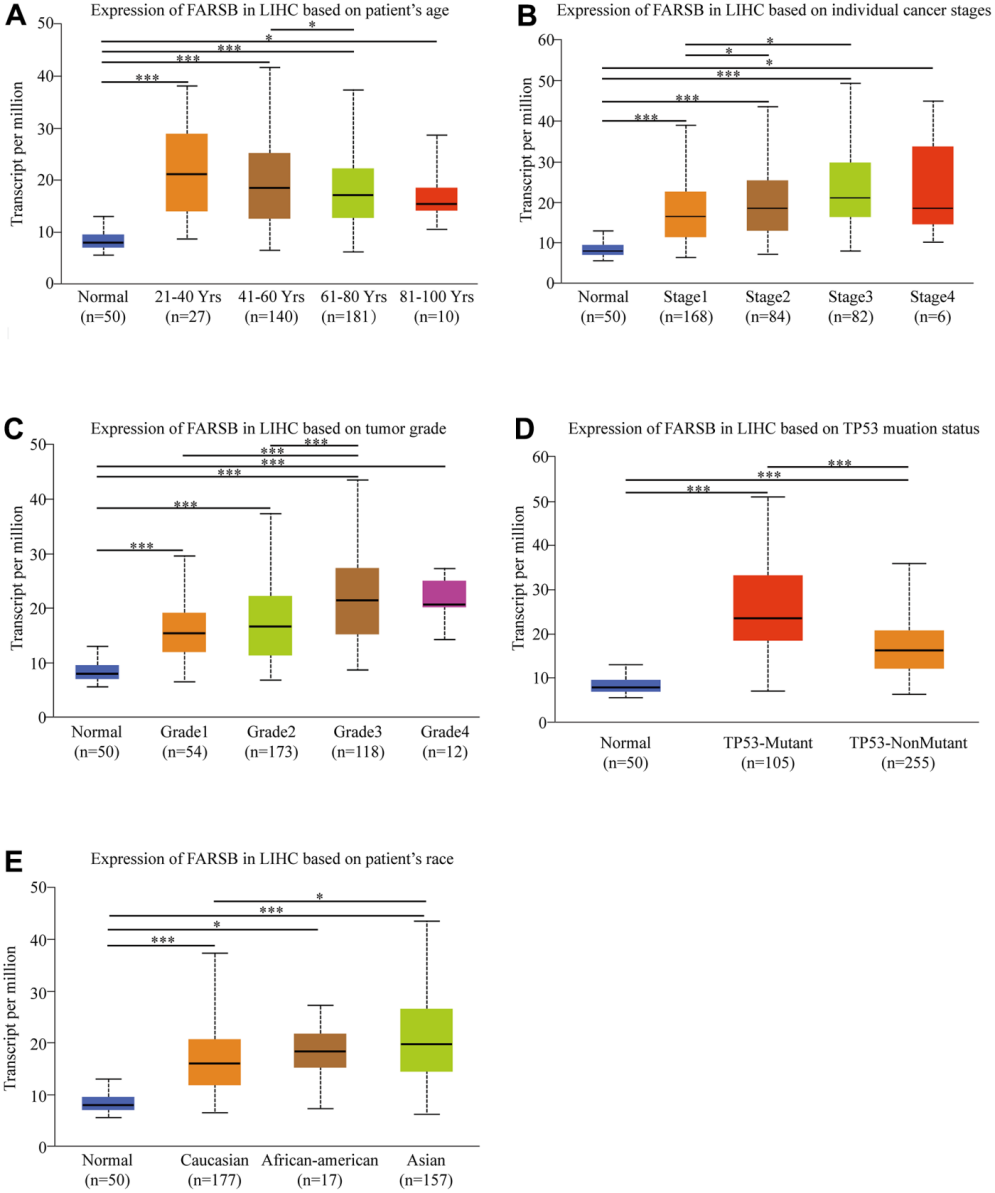
**Box-plots exploring the relationship between FARSB expression and clinicopathological characteristics (UALCAN).** Increased FARSB expression was significantly with (**A**) age, (**B**) cancer stage, (**C**) tumor grade, (**D**) TP53 muation, (**E**) patient race. *P < 0.05; **P < 0.01; ***P < 0.001.

Given the high FARSB expression in HCC patients, for the purpose of finding out the mechanism of FARSB in development of HCC, we utilized the UALCAN online tool to investigate the relationship between clinicopathological features and FARSB expression. The results showed that FARSB expressed significantly different in age, cancer stages, tumor grade, TP53 methylation and race. What’s more, there were significant differences among subgroups (Figure 2A–2E). The expression of FARSB was correlated with clinicopathological features only when the data between subgroups were statistically significant. For instance, FARSB expression level would be higher in stage 3 and 2 than in stage 1 (Figure 2B). Meanwhile, it was also higher in grade 3 than in grade 1 and 2 (Figure 2C). Besides, to analyze the relationship between FARSB expression and clinicopathological variables in greater depth, we utilized logistic regression analysis. The result showed that high FARSB expression was obviously related to age, grade, stage and tumor size (Table 1). It was obvious that FARSB expression was closely related to clinicopathological variables; additionally, FARSB expression was closely related to the stage and grade of indicators that predicted HCC progression. Hence, FARSB had the potential to be a biology marker for screening high risk HCC patients.

**Table 1:** Logistic analysis of the association between FARSB expression and clinical characteristics.

**Clinical characteristics**	**Total (N)**	**Odds ratio in FARSB expression**	***P* value**
**Age** (>60 vs. ≤60)	370	0.66(0.44-1.00)	**0.048**
**Gender** (Female vs.Male)	371	0.81(0.52-1.25)	0.337
**grade**(III+IV vs.I+II)	334	3.45(2.18-5.55)	**<0.001**
**Stage** (III+IV vs.I+II)	347	1.85(1.14- 3.04)	**0.014**
**T** (T3+T4 vs. T1+T2)	368	1.74(1.08-2.82)	**0.023**
**N** (N1 vs. N0)	256	3.05(0.38-62.07)	0.337

T, tumor; N, node; M, metastasis; Bold values indicate *P*-values<0.05.

### Prognostic value of FARSB in HCC

**Figure 3:**
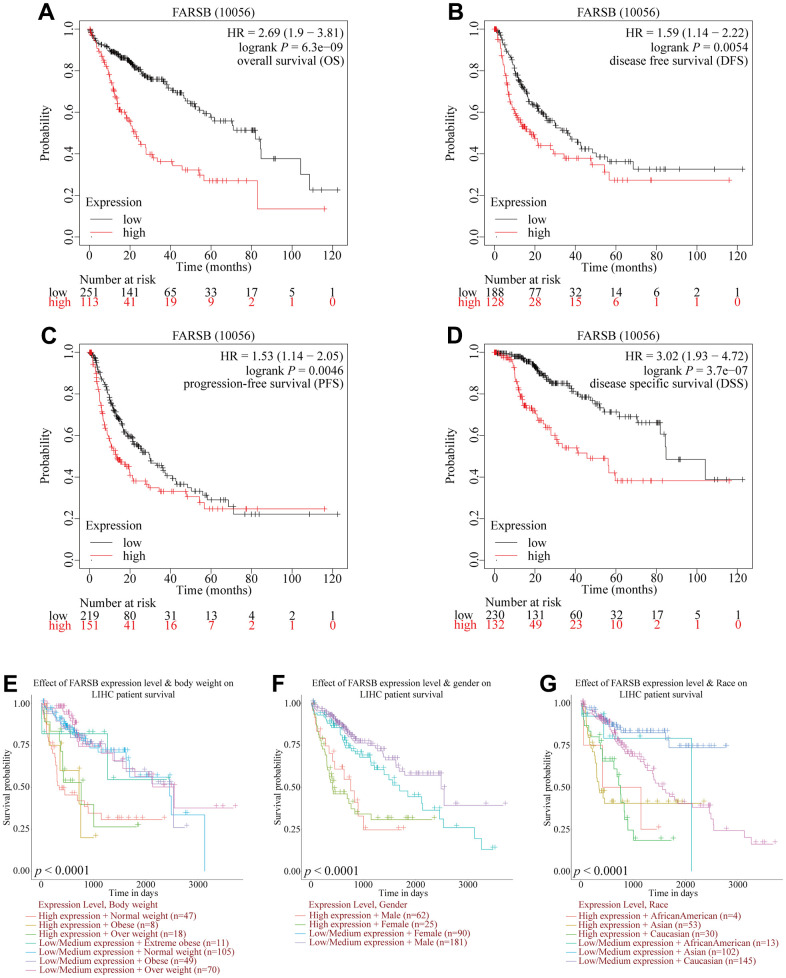
**Comparison of survival curves of FARSB overexpression and underexpression in HCC.** (**A**–**D**) Survival curves for OS, DFS, PFS, and DSS in normal and overall FARSB patients by using Kaplan-Meier Plotter. (**E**–**G**) Internal validation of the prognostic model in TCGA cohort based on clinical features by using UALCAN (**E**) BMI, (**F**) Gender, (**G**) Race.

For the purpose of investigating the link between FARSB expression and HCC prognosis, the Kaplan-Meier Plotter was utilized. And correlational analysis showed that patients who had high-expressed FARSB had short OS, DFS, PFS, and DSS compared with low-expressed FARSB. (Figure 3A–3D, *P*<0.05). Moreover, the UALCAN database results reflected that higher FARSB expression was obviously related with shorter survival of HCC patients in BMI, gender, and race subgroups (Figure 3E–3H, *P*<0.05). These results discovered that high-expressed FARSB is closely related to poor prognosis of HCC.

### Overexpression of FARSB is an independent prognostic factor of HCC

**Figure 4:**
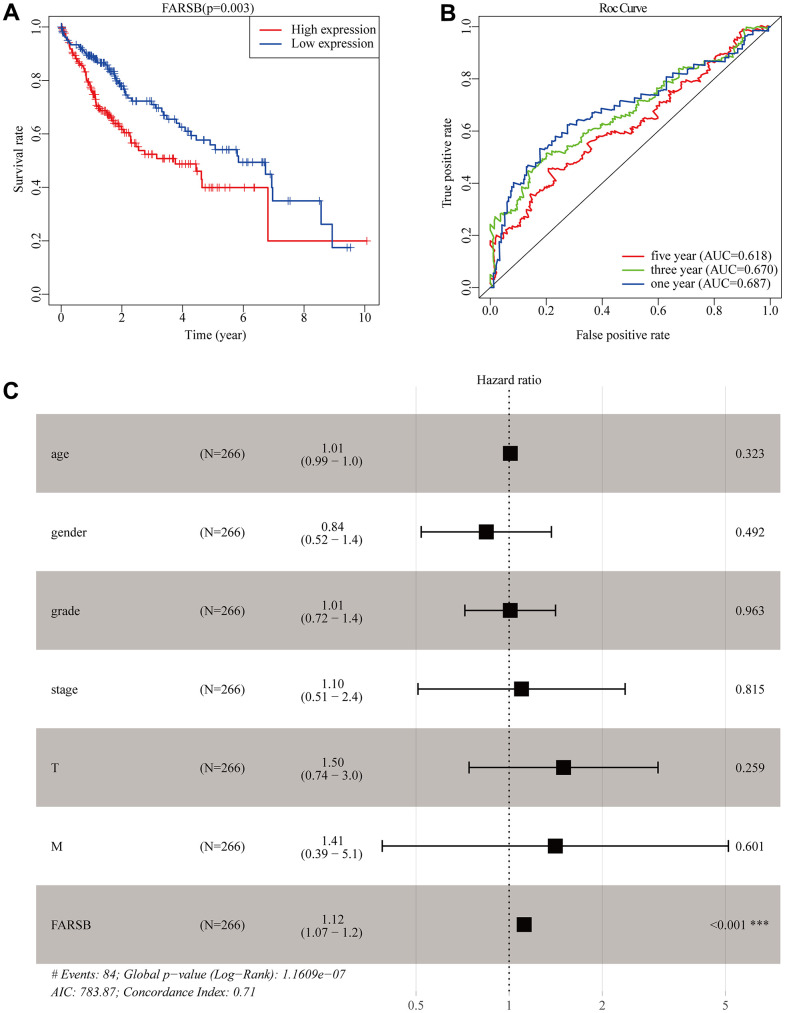
**Construction of the prognostic model in TCGA cohort.** (**A**) The Kaplan-Meier survival analysis for overall survival (OS) of patients in TCGA cohort. (**B**) The time-dependent ROC analysis for risk score in the TCGA cohort. (**C**) Forest plot of the univariate and multivariate Cox regression analysis in HCC regarding OS.

Similar with the results above, the survival curves and ROC curves drawn by R software proved that the high-expressed FARSB was strongly linked with the low overall survival rate, and AUC of the prognosis model at 1, 3 and 5 years was 0.687, 0.670, and, 0.618, respectively, inferring that overexpression of FARSB led to poor prognosis of HCC patients is accurate (Figure 4A, 4B). Then, the univariate and multivariate Cox models were then used to examine the link between clinical characteristics and prognosis in HCC patients. Univariate analysis perform that stage, T stage, and FARSB expression were closely related to worse OS. Multiple-factor analysis reveals that, FARSB expression was found to be an independent prognostic factor (Table 2). These were directly reflected in the forest map (Figure 4C). These results indicated that the overexpressed FARSB was related to poor prognosis so FARSB could independently predict the prognosis of HCC possibly.

**Table 2:** Univariate and multivariate COX regression analysis of factors associated with OS in Liver cancer patients.

**Variable**	**Univariate analysis**				**Multivariate analysis**		
	**HR**	**95%CI**	**P-value**		**HR**	**95%CI**	**P-value**
**age**	1.007	0.990-1.024	0.441		1.009	0.991-1.026	0.323
**gender**	0.839	0.536-1.314	0.443		0.844	0.520-1.369	0.492
**grade**	1.073	0.795-1.449	0.645		1.008	0.719-1.412	0.963
**stage**	1.809	1.426-2.294	**<0.001**		1.096	0.507-2.370	0.815
**T**	1.767	1.415-2.207	**<0.001**		1.499	0.742-3.028	0.259
**M**	3.892	1.223-12.386	0.021		1.410	0.389-5.112	0.601
**FARSB**	1.127	1.078-1.179	**<0.001**		1.118	1.066-1.173	**<0.001**

OS, overall survival; HR, hazard ratio; CI, confidence interval; T, tumor; N, node; M, metastasis; Bold values indicate P-values<0.05.

### The promoter methylation level of FARSB in HCC patients

DNA methylation is one of the essential epigenetic mechanisms. Recent reports have revealed that DNA methylation participates in HCC metastasis and proliferation via epigenetic regulation of oncogenes and tumor suppressor genes.

**Figure 5:**
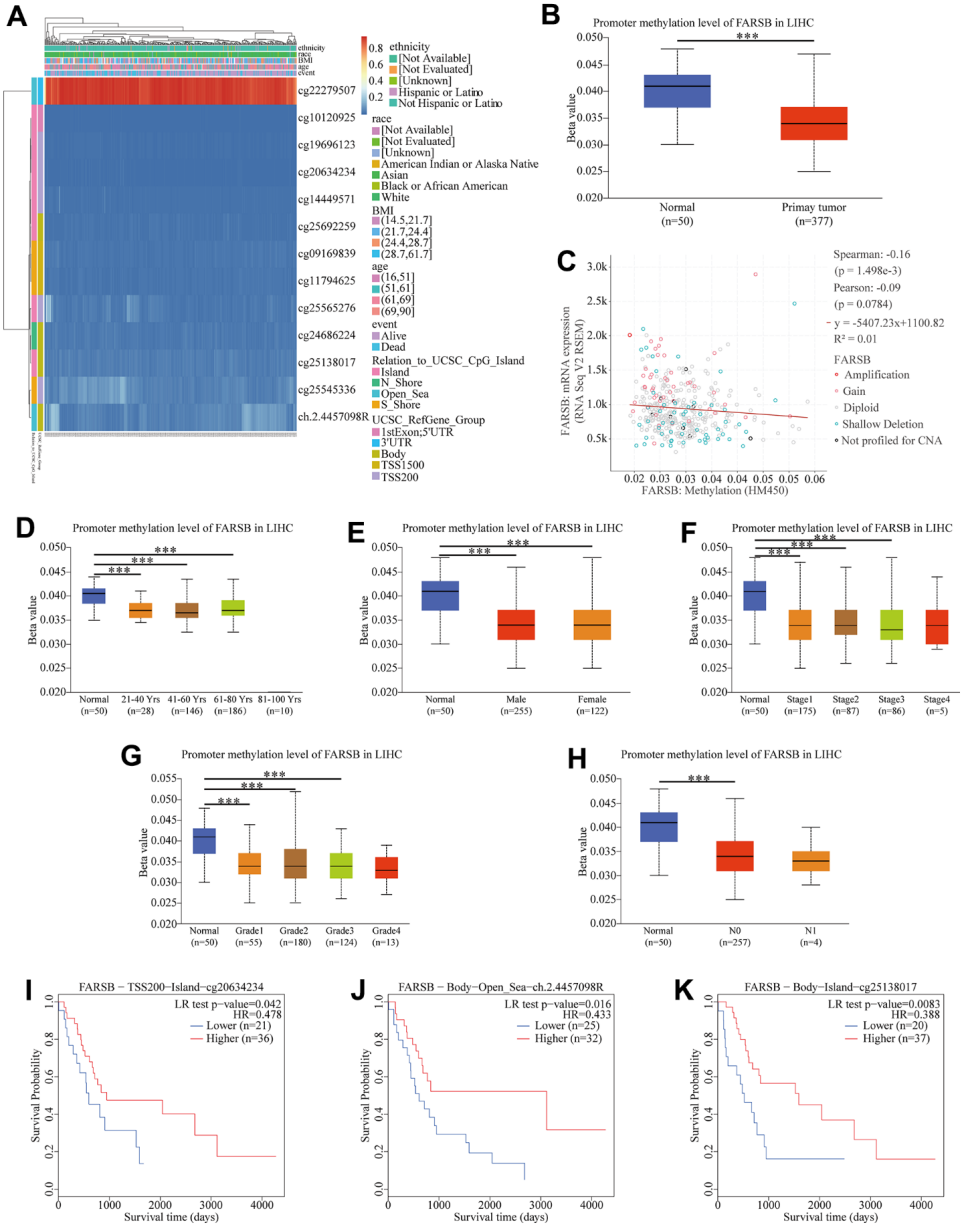
**Correlation between FARSB promoter methylation level and prognostic value of DNA methylation in HCC.** (**A**) Heatmap of relationship between methylation and prognosis in CpG sites. (**B**) Normal vs primary tumor. (**C**) High methylation level of FARSB connected with down-regulated expression. (**D**) Normal vs gender, (**E**) age, (**F**) cancer stage, (**G**) tumor grade, (**H**) correlated with good OS. (**I**) Lymph node metastasis status; *P < 0.05; **P < 0.01;***P < 0.001. High methylation level of cg20634234 (**J**), ch.2.4457098R (**K**), cg25138017.

Therefore, we detected the methylation level of the FARSB promoter in HCC tissues through the MethSurv website. The correlation heat map showed that 12 sites were hypomethylated (Figure 5A). The results of Figure 5A were then verified by the UALCAN database, promoter methylation in HCC tissues were lower than in normal tissues (Figure 5B). Then, we looked into the link between FARSB promoter methylation and FARSB expression levels by cBioporta. The results revealed that FARSB promoter methylation level was inversely linked to FARSB expression. In other words, when FARSB was highly expressed, its promoter was poorly methylated (Figure 5C). Besides, the promoter methylation of the FARSB subgroup was assessed in relation to various clinical characteristics. We discovered that promoter methylation level was negatively connected with patient age, gender, cancer stage, and tumor grade (Figure 5D–5G), but not related to Nodal Metastasis Status (Figure 5H) Moreover, in the heatmap, 3 of the 12 CpG sites linked with hypomethylation were connected with a worse prognosis (P<0.05), including cg20634234, cg25138017, and ch.2.4457098R (Figure 5I–5K). These results testified that the hypomethylation of FARSB is bound up with a poor prognosis in HCC.

### In HCC, the gene most associated with FARSB and cellular pathway that FARSB participate

**Figure 6:**
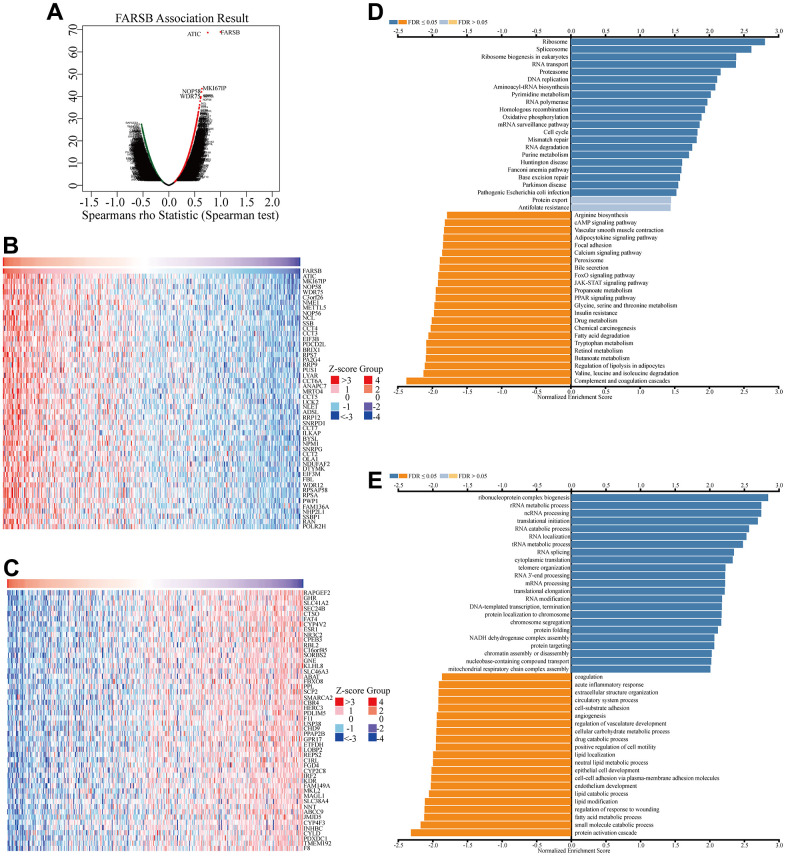
**Co-expression genes of FARSB in HCC.** (**A**) Volcano plot of genes highly correlated with FARSB identified by the Spearman test in LIHC. Red and green dots represent genes significantly positively and negatively correlated with FARSB, respectively. Heatmaps of the top 50 genes (**B**) positively and (**C**) negatively correlated with FARSB. (**D**, **E**) Significantly enriched GO and KEGG pathways of FARSB. GO: Gene Ontology; KEGG: Kyoto Encyclopedia of Genes and Genomes.

Given that FARSB expression was markedly correlated with the HCC prognosis, we tried to find out the pathway in which FARSB was involved in order to better understand the biological functions of FARSB in HCC. To start with, we utilized “LinkFinder” in LinkedOmics to explore FARSB co-expression genes in HCC. According to the Spearman test, there were 9879 genes (dark red dots) that had a positive correlation with FARSB, while 10042 genes (dark green dots) had a negative connection (false discovery rate, FDR<0.01) (Figure 6A). Next, a heatmap depicted the top 50 genes that were positively and negatively linked with FARSB (Figure 6B, 6C). Following that, KEGG pathway analysis showed that it was rich in cell cycle, DNA replication, and other processes. (Figure 6D). And the GO term annotation discovered that the FARSB co-expressed genes were primarily involved in DNA-templated transcription, among other things (Figure 6E).

**Figure 7:**
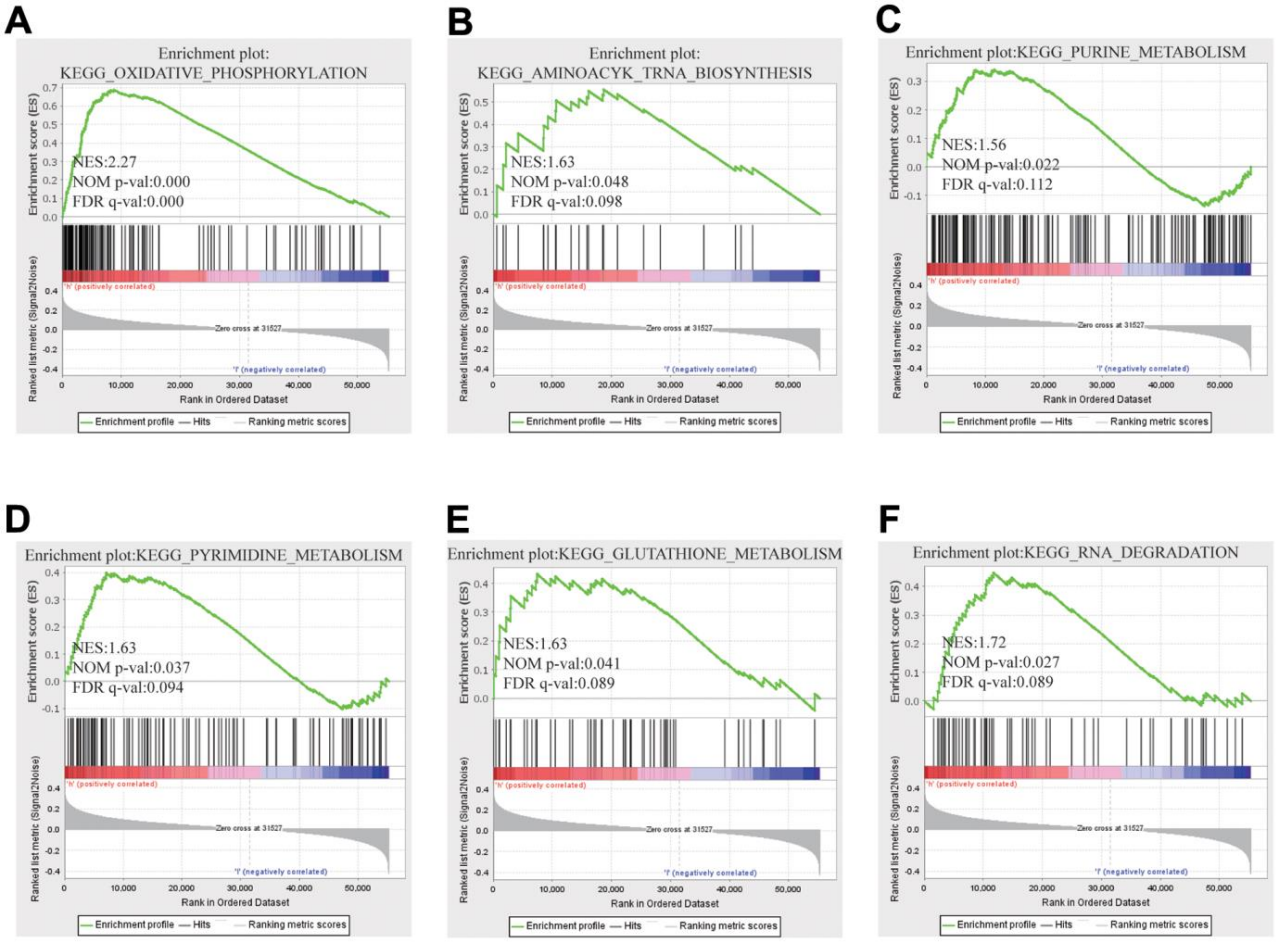
**GSEA results showed differential enrichment of genes with high FARSB expression.** GSEA was used to validate the gene signatures, including positive regulation of (**A**) oxidative phosphorylation, (**B**) aminoacyl tRNA biosynthesis (**C**) purine metabolism, (**D**) pyrimidine metabolism and (**E**) Gglutathione metabolism, (**F**) RNA RNA degradation.

In contemplation of investigating underlying regulatory pathway of FARSB in HCC, we chose the higher and lower FARSB expression groups to display GSEA analysis. Some important pathways, like oxidative phosphorylation and otherwise some metabolism pathways such as purine metabolism, pyrimidine metabolism and glutathione metabolism have been found. Also, RNA-related pathway, RNA degradation and biosynthesis of aminoacyl tRNA were discovered (Figure 7A–7F).

### Associations between FARSB and immune infiltration in HCC

**Figure 8:**
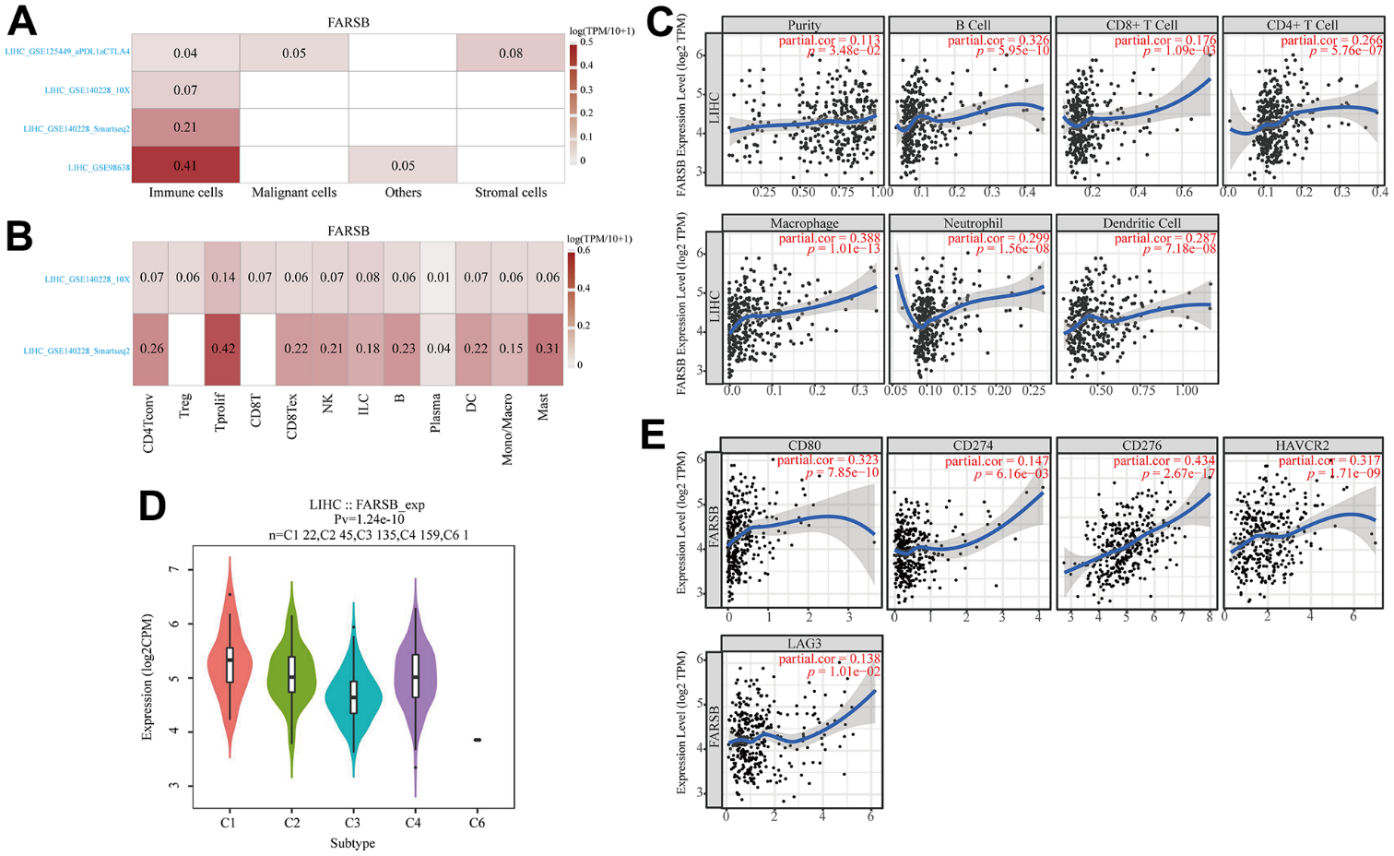
**Associations between FARSB and immune infiltration in HCC.** (**A**) FARSB expression in HCC tissues. (single-cell sequencing) (**B**) FARSB expression in immune cell in HCC. (single-cell sequencing) (**C**) FARSB expression in HCC tissues positively correlates with the tumor purity (r=0.113, *P*=3.48e-02) and infiltration levels of B cells (r=0.326, *P*=5.95e-10), CD8+ T cells (r=0.176, *P*=1.09e-03), CD4+ T cells (r=0.266, *P*=5.76e-07), Macrophages (r=0.388, *P*=1.01e-13), Neutrophils (r=0.299, P=1.56e-08), and DCs (r=0.287, *P*=7.18e-08) in HCC tissues. (**D**) Distribution of FARSB expression across immune subtypes in HCC (TISIDB). The different color plots represent the five immune subtypes (C1: wound healing; C2: IFN-gamma dominant; C3: inflammatory; C4: lymphocyte-depleted and C6: TGF-b dominant). (**E**) FARSB expression in HCC tissues significantly correlates with T cell checkpoints. (CD80 (r=0.323, *P*=7.85e-10), CD274 (r=0.147, *P*=6.16e-03), CD276 (r=0.434, *P*=2.67e-17), HAVCR2 (r=0.317, *P*=1.71e-09), LAG3 (r=0.138, *P*=1.01e-02), PDCD1 (r=0.237, *P*=8.41e-06)).

To lucubrate the mechanism of FARSB in hepatocellular carcinoma, scRNA-seq was used to detect the expression of FARSB in different cell types of HCC. The result demonstrated that FARSB was highly expressed in immune cells group in all LIHC data sets (Figure 8A). Then, we investigated the link between FARSB expression and the infiltrating immune cells (hepatocellular carcinoma) by utilized TIMER “Gene” module. According to this graph FARSB expression was positively related to the tumor purity and infiltration of some immune cells (Figure 8B, P< 0.05). In order to find which type of immune cells that FARSB was mainly expressed, researcher tested the expression of FARSB in various immune cells by scRNA-seq. This figure suggests that FARSB was highly expressed in T cells (Figure 8C).

Additionally, TISIDB was used to detect FARSB expression in various immune subgroups in HCC. Our team defect FARSB express highly in C1 and C2 clusters and low in C6 (Figure 8D). We also looked into the connection between FARSB expression and T cell checkpoints (such as PD1, PD-L1, LAG3, CD80, B7-H3, PD-L2, IDO1, and TIM-3) through the GEPIA database (Table 3). The expression of FARSB has a significant relationship with PD-1, PD-L1, LAG3, CD80, B7-H3, and TIM-3 in HCC (Figure 8E). Altogether, the data suggested that FARSB expression is intimately related to the degree of the infiltration of T cells.

**Table 3:** Correlation analysis between FARSB and immune checkpoint of T cells in TIMER.

**Immune checkpoint**	**LIHC**			
	**None**		**Purity**	
	**Cor**	**p**	**Cor**	**p**
LAG3	0.116249475	**2.51E-02**	0.138354618	**0.010085575**
HAVCR2 (TIM-3)	0.219261558	**2.04E-05**	0.317018555	**1.71E-09**
CD274 (PD-L1)	0.119925938	**2.09E-02**	0.147204223	**6.16E-03**
CD276 (B7-H3)	0.430900746	**3.32E-18**	0.434263139	**2.67E-17**
CD80	0.246025326	**1.61E-06**	0.323238056	**7.85E-10**
PDCD1LG2 (PD-L2)	0.024668832	6.36E-01	0.082462065	0.126336179
PDCD1 (PD-1)	0.168691211	**1.11E-03**	0.237248349	**8.41E-06**
IDO1	0.050914859	3.28E-01	0.071472885	1.85E-01

### Prognostic analysis of FARSB expression in HCC in the view of immune cells

**Figure 9:**
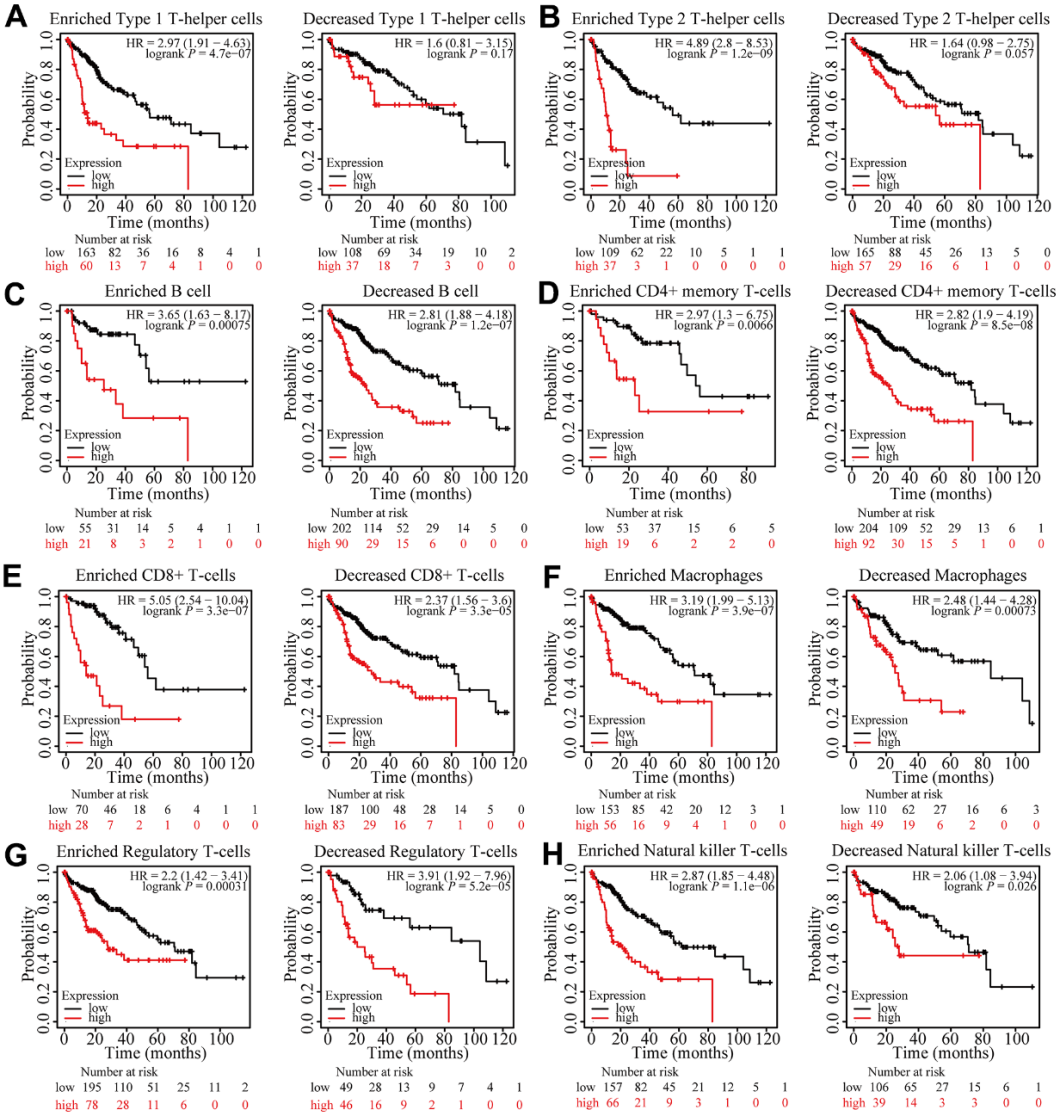
**Prognostic analysis of FARSB expression in HCC in view of immune cells.** Kaplan-Meier survival curves according to high and low expression of FARSB in immune cell subgroups in HCC. (**A**–**H**) Correlations between FARSB expression and OS in different immune cell subgroups in HCC patients.

According to the results above, FARSB is attributed with immune infiltration in HCC. Moreover, we looked into whether FARSB expression affects the prognosis of HCC caused by immune invasion. The Kaplan-Meier Plotter had been utilized to conduct a prognostic evaluation for patients in different FARSB expression levels across various immune cell subsets. We observed that FARSB expression didn’t show any obvious difference in the prognosis of HCC under different infiltration levels of these immune cells (Figure 9C–9H). However, high-expressed FARSB was linked with a poor prognosis in enriched Th1 and Th2, while there was no strong association between its decreased subgroups (Figure 9A, 9B). Therefore, our conjectural theory is FARSB may affect the prognosis of HCC by enriching Th1 and Th2. The high expression of FARSB may constitute an immunosuppressive microenvironment by affecting the expression levels of relevant chemokines, CCL26 and CX3CL1, and helping the infiltration of Th2 cells, resulting in a poor prognosis for liver cancer patients.

These findings indicated that FARSB may affect the prognosis of those who suffer from hepatocellular carcinoma by recruiting Th1 and Th2 to influence immune infiltration. Overall, our study further confirmed that FARSB could make a difference in HCC’s development. It also leads to poor prognosis by modulating immune infiltration.

### Relationship between FARSB expression and m6A modification in HCC

N6-methyladenosine (m6A) is the most common modification of eukaryotic RNAs which is required for a number of biological processes. Previous research has verified that abnormal regulation of m6A modification is linked to a wide range of cancer in different human organs, such as lung, breast and liver.

**Figure 10:**
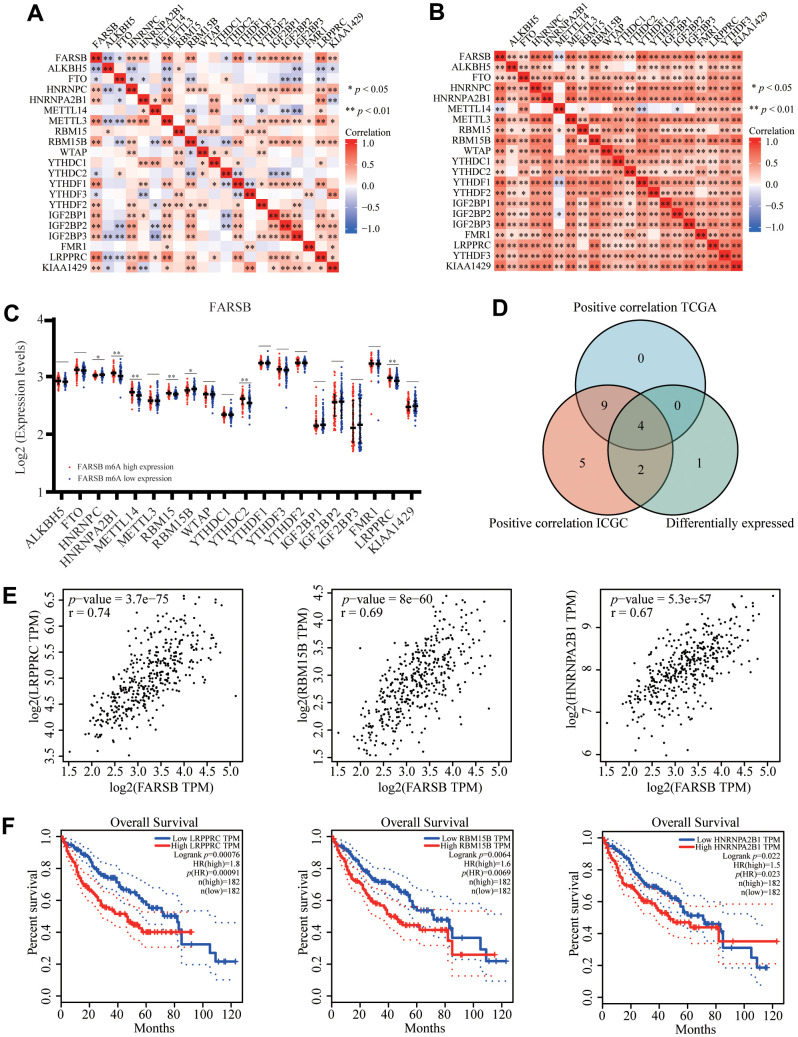
**Correlation of FARSB expression with m6A related genes in HCC.** (**A**, **B**) TCGA HCC data set and ICGC data set analyzed the correlation between the FARSB and m6A related genes expression in HCC. (**C**) The differential expression of glycolysis related genes between high and low FARSB expression groups in HCC tumor samples. (**D**) Venn diagram showed both expression correlation and differential expression of genes including LRPPRC, RBM15B, HNRNPA2B1, HNRNPC. (**E**) A scatter plot was drawn to show the correlation between the FARSB and m6a related genes expression, including LRPPRC, RBM15B, HNRNPA2B1, HNRNPC. (**F**) Kaplan-Meier curve of LRPPRC, RBM15B, HNRNPA2B1 *P < 0.05; **P < 0.01; ***P < 0.001.

By analyzing TCGA and ICGC HCC data, we detected a link between FARSB expression and twenty-one m6A interrelated genes expression of HCC. In the TCGA database, FARSB expression was correlated positively with LRPPRC, RBM15B, HNRNPA2B1, and YTHDF1 (Figure 10A, *P*<0.01). Furthermore, FARSB expression was correlated positively with RBM15B, LRPPRC, YTHDF1, HNRNPC, and HNRNPA2B1 in ICGC data sets (Figure 10B, *P* < 0.01). We divided TCGA samples into two groups according to the expression of FARSB. We tried to compare the expression of genes involved in m6A modification between the two groups. As shown in Figure 10, the m6A modification was not the same in high and low groups with the FARSB expression in HCC (Figure 10C). Compared to the group of low expression, the expression of 7 genes in the FARSB high expression group was increased (*P* <0.05). Both expression correlation and differential expression of genes, were presented in Venn’s diagram, including LRPPRC, RBM15B, HNRNPA2B1, and HNRNPC. (Figure 10D). The scattering plot shows the relationship between FARSB and m6A related genes expression (Figure 10E) Then, we used the Kaplan-Meier curve to reveal that high expression of LRPPRC, RBM15B, and HNRNPA2B1 was intensely associated with a poor prognosis of HCC (*P*<0.001) (Figure 10F). These results claim that, in HCC, FARSB may have a close relation with the m6A modification, specifically via its interactions with LRPPRC, RBM15B, and HNRNPA2B1, all of which eventually influence the progression and prognosis of HCC.

### FARSB-related ceRNA regulatory network

In recent year, many research articles highlighted the regulatory role of lncRNA-miRNA-mRNA ceRNA networks in cancers. Hence we established a FARSB ceRNA regulatory network in HCC.

**Figure 11:**
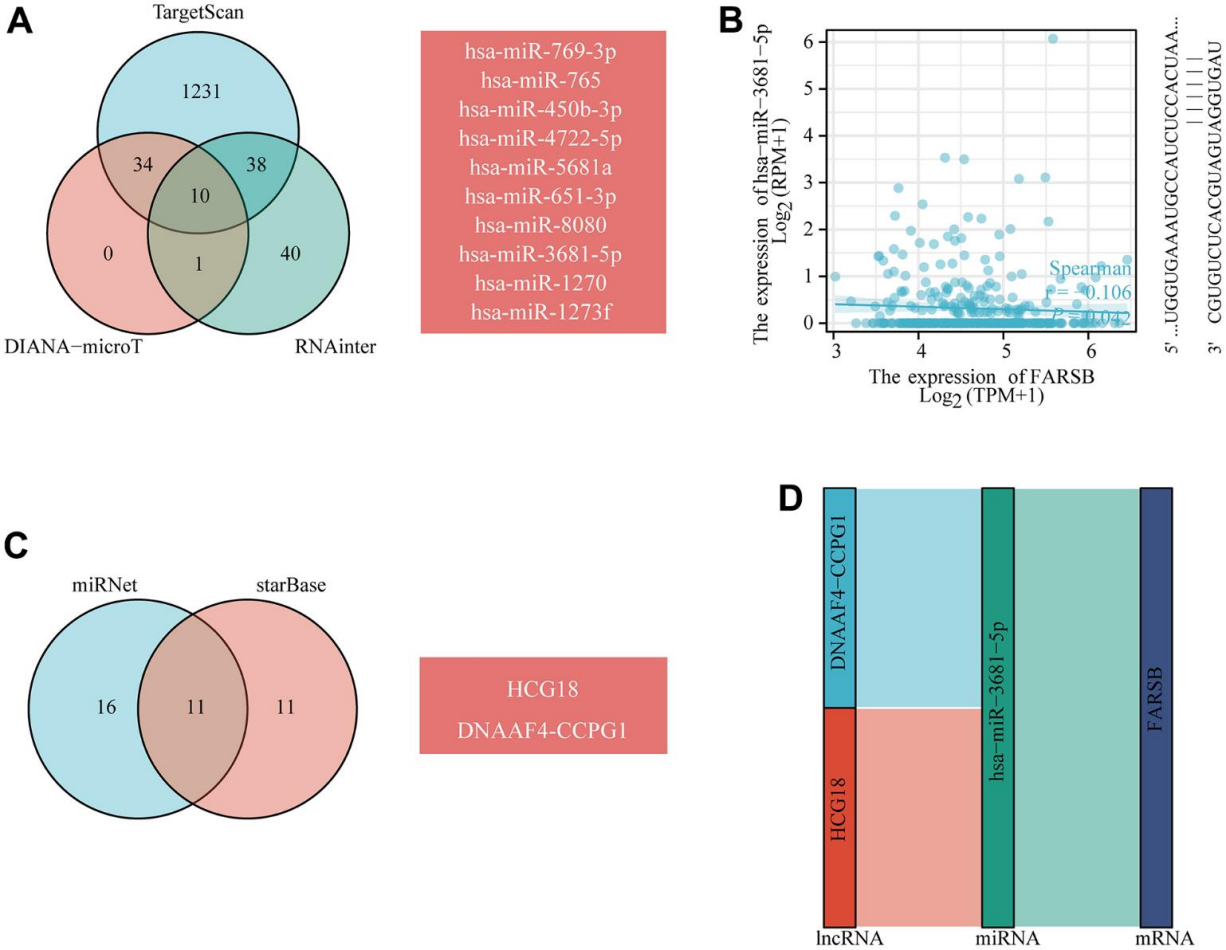
**Prediction of the ceRNA network in HCC.** (**A**) Venn diagram showing the results for FARSB targets predicted using the TargetScan, DIANA-microT and RNAinter databases. (**B**) Scatter plots were generated to show miRNAs-mRNAs with significant correlations. (**C**) The lncRNAs that bind to target miRNAs were predicted using the miRNet and starBase online databases and displayed in a Venn diagram. (**D**) Sankey diagram showing the FARSB-related ceRNA regulatory network.

TargetScan, DIANAmicroT and RNAinter database predictions all predicted the following 10 miRNAs: hsa-miR-769-3p, hsa-miR-765, hsa-miR-450b-3p, hsa-miR-4722-5p, hsa-miR-5681a, hsa-miR-651-3p, hsa-miR-8080, hsa-miR-3681-5p, hsa-miR-1270, and hsa-miR-1273f (Figure 11A). According to the ceRNA control correlation, the relationship of mRNAs and miRNAs was proven to be a negative correlation. Through correlation analysis, Hsa-miR-3681-5p was negatively correlated with FARSB expression (Figure 11B). Then, the lncRNAs which perhaps interact with hsa-miR-3681-5p were forecast by the miRNet and starBase databases (Figure 11C). As of now, science research has confirmed a negative correlation between the expression of lncRNAs and miRNAs. Hence, the starBase database was utilized to find the lncRNAs that were negatively related with hsa-miR-3681-5p in HCC. Eventually, two ceRNA regulatory pathways that may exist in HCC were established: HCG18-hsa-miR-3681-5p-FARSB, and DNAAF4-CCPG1-hsa-miR-3681-5p-FARSB (Figure 11D). This suggested to us that the HCG18/DNAAF4-CCPG1-hsa-miR-3681-5p-FARSB axis may regulate FARSB expression in HCC.

### Cancer pathway activity and drug sensitivity

**Figure 12:**
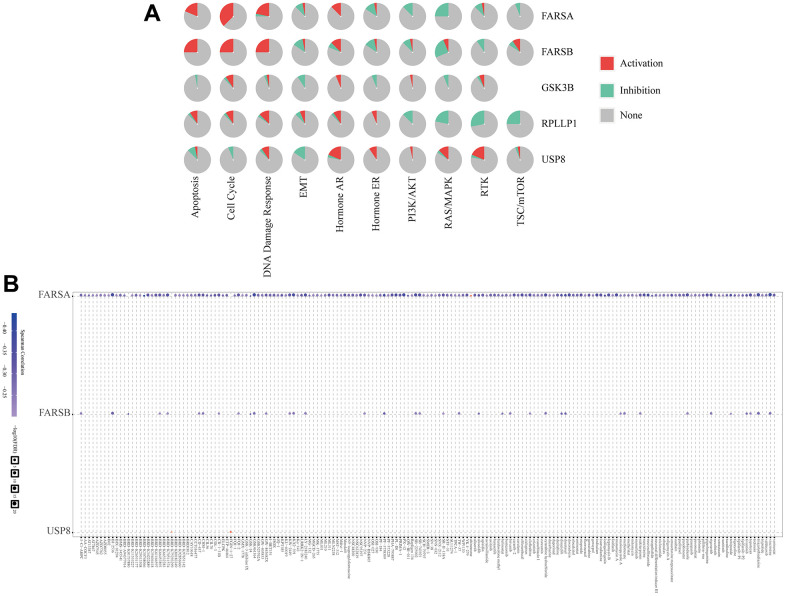
**The correlation of drug sensitivity and cancer pathway activity about FARSB in HCC patients.** (**A**) FARSB-related cancer pathway activity. (**B**) FARSB-related drug sensitivity using GDSC.

Based on the above results, we used the GSCALite tool to evaluate the possible role of the top five GeneMANIA selected genes in the classical cancer pathways. As shown in our results, these genes, especially FARSB, could activate Cell Cycle, DNA Damage Response, Hormone AR pathways, TSC/mTOR and inhibit EMT, Hormone ER, PI3K/AKT, RAS/MAPK, RTK pathways to play a regulatory role in the cancer process (Figure 12A). In addition, the cell with high-expressed FARSB were sensitive to 38 drugs or small molecules (Figure 12B). These findings showed novel and selectable treatment options for HCC patients with high FARSB expression.

## DISCUSSION

Hepatocellular carcinoma (HCC), the main pathological histological type of primary liver cancer, is one of the most common malignancies today. And it’s the leading cause of cancer-related deaths worldwide. Early detection and treatment is an effective means of improving survival in patients with hepatocellular carcinoma. However, the most commonly used biomarker for HCC detection, alpha-fetoprotein (AFP), still shows low sensitivity and heterogeneous specificity at various cut-off points. Identification of useful biomarkers for monitoring HCC remains inadequate [42]. It is therefore of interest to explore more biomarkers for the post-treatment prognosis of HCC, and they may have more clinical utility in the near future. In our article, we combined bioinformatic analysis and *in vitro* experiments to analyse the expression of FARSB in HCC. We also further surveyed its connection with immune infiltration, m6A modification and drug sensitivity.

In our research, we first identified elevated mRNA levels of FARSB in hepatocellular carcinoma compared to normal tissues through the Timer online website. Immediately after we verified the results of significantly higher FARSB mRNA expression using TCGA, ICGC, and GEO databases, we next examined FARSB expression at the protein level in clinical samples. Our research exposed that FARSB was highly expressed in hepatocellular carcinoma. In conclusion, we noticed that FARSB was highly expressed in HCC at both mRNA and protein levels, using public databases and HCC clinical samples.

After discovering the close relationship between expression and clinicopathological features, we then went on to discover the prognostic value of FARSB expression in hepatocellular carcinoma. Kaplan-Meier Plotter survival analysis exhibited that high FARSB expression had correlation with four types of poor survival including OS, DFS, PFS, and DSS. Next the overall survival OS of patients at high FARSB expression was validated using R and the results showed that patients with high FARSB expression had lower overall survival. ROC curve analysis presented that FARSB expression had predictive value for the evaluation of survival in HCC patients. We then further explored the prognostic value of FARSB expression in hepatocellular carcinoma using single multifactor Cox analysis, and the results showed that FARSB could be considered as an independent predictor.

More and more studies have shown that epigenetic modification can affect the accumulation of genetic changes during the development of liver cancer [43–47], in which abnormal DNA methylation has been detected could gradually increase with the progression of cancer [48]. Therefore, to further investigate the possible mechanism of FARSB overexpression in liver cancer, we studied the methylation of FARSB promoter in liver cancer, and observed that in HCC patients, the degree of promoter methylation was negatively correlated with FARSB expression. In addition, our analysis exposed that the FARSB promoter was poorly methylated in HCC and correlated with clinical stage, histological grade, lymph node metastasis, age, and gender. Meanwhile, the FARSB promoter is hypomethylated at many CpG sites and is related to poor prognosis. These studies suggest that the high expression of FARSB in HCC may be due to hypomethylation of FARSB promoter, and both high expression of FARSB and hypomethylation predict poor prognosis in HCC patients.

In order to further explore the biological function and potential molecular mechanism of FARSB expression affecting the occurrence and development of HCC, we analyze the enrichment of genes significantly related to FARSB expression in HCC. FARSB may form a regulatory network with these genes to promote the occurrence and development of HCC. GO and KEGG results showed that the biological functions of FARSB were associated with cell cycle, DNA replication, acute inflammatory response and base excision repair. Some genes in the regulatory network have been confirmed to affect the occurrence and development of HCC through cell cycle. For example, WDR4 promotes HCC cell proliferation by inducing G2/M cell cycle conversion and inhibiting apoptosis [49], while DTYMK up-regulation enhances HCC growth and proliferation by promoting cell cycle [50]. GSEA enriched these genes co-expressed with FARSB and found that this regulatory network could promote oxidative phosphorylation, base resection and repair, pyrimidine and purine metabolism, and inhibit cell apoptosis. When FARSB is highly expressed, “oxidative phosphorylation”, “base resection and repair”, “pyrimidine metabolism, purine metabolism” and other pathways are significantly up-regulated, while “apoptosis” pathway is significantly down-regulated, which is consistent with the characteristics of liver cancer progression. Changes in liver metabolism are critical to the development of liver disease, and mitochondrial oxidative phosphorylation contributes to the development or progression of hepatocellular carcinoma [51]. Meanwhile, inhibition of apoptosis has also been proved to be an important factor in the progression of HCC [52], for example, inhibition of apoptosis induced can promote the progression of HCC [53]. In conclusion, the signaling pathways associated with high expression of FARSB can promote the progression of cancer. Whether FARSB can promote the development of HCC by regulating cell cycle, oxidative phosphorylation and apoptosis signaling pathways remains to be further studied.

Recently, a large number of studies have proved that immune microenvironment (TME) takes a crucial part in tumor genesis and development [54, 55], and immune cell infiltration is closely associated with tumor tissue formation, survival and metastasis [56]. TGF-β derived from TME in HCC can promote the expression of tim-3 in tumor-associated Macrophages (TAMs). Activated TAM promotes tumor growth and immune tolerance through NF-κB/IL-6 pathway [57]. At the same time, tumor immune cell infiltration can also influence the prognosis of cancer patients and the efficacy of immunotherapy [58, 59]. For example, in HCC, intertumor neutrophil infiltration indicates a poor prognosis associated with CXCL5 overexpression [60]. To further understand the role of FARSB in HCC, we investigated the association between FARSB gene expression and immune cells. First, we detected the expression of FARSB in a variety of immune cells. Next, we continued to explore the connection between the expression of FARSB and immune cell infiltration, and the findings displayed that the expression level of FARSB was related to many kinds of immune cells in HCC. Our result showed that FARSB may take a certain part in the immune infiltration of HCC.

With a better understanding of mechanisms of cancer progression, we have discovered more ways to treat tumors [61], among which immune checkpoint blockade (ICB) has been a great success and immune checkpoint inhibitors (ICI) are used as first-line therapy for advanced HCC, such as inhibition of CTLA-4 and PD-1 expression. Immunotherapies that inhibit the expression of CTLA-4 and PD-1 have been effective in the treatment of various tumors [62–64], and tumor immunotherapy requires sufficient immune cells to infiltrate the tumor microenvironment and sufficient immune checkpoint expression to achieve efficacy [65]. Currently, only 10-20% of the population benefit from immunotherapy [66]. Therefore, it is of great interest to discover more new biomarkers to improve prognosis and individual tumor treatment. Considering that FARSB is highly correlated with HCC immune cell infiltration, to continue to understand the contribution of FARSB in HCC immunotherapy, we revealed the relationship between FARSB and HCC immune checkpoints, and we explored that high expression of FARSB was closely associated with PD1, PD-L1, CTLA-4, CD80, B7-H3, and TIM-3. These results revealed that patients with high FARSB expression may benefit from immunotherapy for HCC, and that rational application of CTLA-4 and PD-1 or its ligands CD80 and PD-L1 inhibitors will facilitate to restore anti-tumor immune responses, which in turn will provide long-term benefits to patients. To explore in depth the role of FARSB in tumor immunity, we investigated the correlation between FARSB and immune cell biomarkers in HCC. The results present an obvious positive relationship between FARSB expression and biomarkers of T cells which further demonstrates a positive correlation between FARSB and immune cell infiltration.

The continuous injury and regeneration of hepatocytes can be caused by the accumulation of immune cells to tumors which can also promote the development of HCC [67–69]. Chemokines take an important part in the recruitment and activation of immune cells and also participate in tumor progression, invasion and metastasis [70, 71]. Interestingly, we noticed that FARSB expression was positively related to chemokines CCL26, CX3CL1 and CCR8, a key surface molecule of TH2 cells, which played a role in chemotactic TH2 cells in HCC [72, 73], suggesting that FARSB expression had close connection with TH2 cell infiltration in HCC. At the same time, studies have shown that different molecular mechanisms affect the progression of liver cancer by triggering characteristic responses in specific immune cell subsets. For example, TH2 cytokines can promote the spread and metastasis of cancer cells in various cancers [74]. An imbalance in the TH1/TH2 cell ratio have relations with reduced survival rate in patients with breast, melanoma, esophageal, and colon cancers [75]. It has also been reported that TH1/TH2 immune cell balance is vital in tumorigenesis and progression [76], and therapies related to regulation of TH1/TH2 balance may have significant implications for cancer immunotherapy. In conclusion, TH1 and TH2 cells take an essential part in the development, prognosis and immunotherapy of cancer. It is worth noting that our research exhibited that the difference in FARSB expression level would lead to the difference in overall survival of patients only when TH1 and TH2 were enriched. Therefore, the high expression of FARSB may constitute an immunosuppressive microenvironment by affecting the expression levels of relevant chemokines, CCL26 and CX3CL1, and helping the infiltration of Th2 cells, resulting in a poor prognosis for liver cancer patients. At the same time, this implies that FARSB may be a new immune-related therapeutic target in HCC.

N6-methyladenosine (m6A) is the most significant mRNA [77], involved in the pathogenesis of many diseases, including cancer, many physiological and pathological processes play an important role [78], a growing body of evidence suggests that RNA N6-methyladenosine (m6A) takes a significant part in proliferation, differentiation, tumor invasion and metastasis [79–81]. However, the relationship between FARSB and m6A modification has not been reported. Our results show that there is a certain correlation between FARSB and m6A modification. Seven M6A-related genes were differentially expressed when FARSB was overexpressed and underexpressed. Four genes most related to FARSB expression were screened out by Venn diagram, including LRPPRC, RBM15B, HNRNPA2B1 and HNRNPC. In addition, by constructing Kaplan-Meier curves, we found that patients with high expression of LRPPRC, RBM15B and HNRNPA2B1 had shorter survival times compared to patients with low expression. Although HNRNPC is positively correlated with FARSB expression, differential expression of HNRNPC gene does not affect the survival of HCC patients, as shown in Supplementary Figure 4, so we speculate that differential expression of HNRNPC gene has no significance in prognosis of patients. Among them, studies have shown that LRPPRC can promote G1/S conversion and cell proliferation in hepatocellular carcinoma [82]. RBM15B has been identified as an independent prognostic indicator of melanoma [83]. HNRNPA2B1 can promote the progression of esophageal cancer by up-regulating fatty acid synthase as a carcinogen [84]. In conclusion, our results suggest that the poor prognosis of patients with higher expression of FARSB may be connected to m6A modification. And FARSB may influence the methylation level of HCC mRNA through its connection with LRPPRC, RBM15B, and HNRNPA2B1, ultimately leading to poor prognosis of HCC patients. Subsequently, we created a ceRNA regulatory network based on prediction. Since FARSB’s ceRNA regulatory network is derived from bioinformatics analysis, we need more experiments to confirm this network in future studies.

With the progress of molecular biology, more and more attention has been paid to the development of HCC treatment drugs and their clinical benefits. It has been reported that advanced HCC patients benefit from tivantinib treatment [85]. However, the limitations of drug therapy such as moderate efficacy of targeted drugs for liver cancer, lack of therapeutic response biomarkers and susceptibility to drug resistance still exist [86]. In our study, HCC cell lines were sensitive to 38 drugs or small molecules when FARB is highly expressed. In particular, HCC cell lines with high expression of FARSB are sensitive to tivantinib, which may provide a new treatment option for HCC patients with high expression of FARSB.

The interaction of protein is usually necessary to implement biological function and metabolic reactions, and protein-protein interaction by the regulation of modification after translation [87]. Therefore, we used the GeneMANIA database to establish an interaction network between FARSB and other tumor-related proteins. The interaction network showed that FARSB could directly interact with RPLP1. Ribosomal protein LP1 (RPLP1) is a member of the Ribosomal protein L12P family [88], which takes an oncogene part in hepatocellular carcinoma [89]. Next, we continued to study the secondary structure of FARSB and RPLP1, and found that FARSB and RPLP1 have multiple modification sites, which can be modified after translation. These results suggest that targeted protein modifications can alter the expression of FARSB and RPLP1, and FARSB may act by interacting with RPLP1. Finally, to further explore the mode of direct interaction, we forecasted encouraging binding sites by a molecular docking model. Our result could furnish a basis for future experimental studies.

In conclusion, our study found hypomethylation and overexpression of FARSB in HCC, which was substantially connected to clinicopathological features and poor prognosis of HCC patients. FARSB expression affected cell cycle and immune microenvironment of HCC. And up-regulation of FARSB expression promoted tumor immune cell infiltration and checkpoint expression. This suggests a new direction for tumor immunotherapy in patients with high expression of FARSB, and is closely related to m6A modification and drug sensitivity. This suggests that FARSB can be used as a diagnostic marker and immune-related therapy target, with an opportunity to refresh diagnostic and treatment choices of HCC.

## Supplementary Material

Supplementary Materials

Supplementary Figures

Supplementary Tables
