# Supplementary material for: FARSB serves as a novel hypomethylated and immune cell infiltration related prognostic biomarker in hepatocellular carcinoma
Source: Aging (Albany NY). 2023 Apr 3;15(8):2937–69. doi: 10.18632/aging.204619 (PMC10188347; doi:10.18632/aging.204619)
Supplement: Supplementary Materials [file aging-15-204619-s001.pdf]

# SUPPLEMENTARY MATERIALS

## Correlation of FARSB expression with cell cycle

In view of the results above, we speculated that FARSB was related to the cell cycle. So STRING database was performed on top 500 co-expressed genes to make the PPI network, and Cytoscape was applied for finding the critical module, which we marked in yellow (Supplementary Figure 1A). The MCODE score for the genes with the highest scores consisted of CDCA3, NCAPG, KIF2C, CCNB1, PTTG1, and CDC20 (Supplementary Figure 1B). And we investigated that there existed an obvious correlation coefficient between FARSB and these genes through GEPIA analysis (Supplementary Figure 1C, 1E). What's more, we have done prognosis analysis of these genes by the Kaplan-Meier Survival Method, which showed that all of these 6 genes were oncogenes that were related to poor prognosis (Supplementary Figure 1D, 1F). The results of pathway analysis proved that all 6 genes in the module were related to the cell cycle in HCC, and based on the above analysis, we inferred that FARSB might be connected to the cell cycle.

## Correlation analysis of FARSB mRNA expression levels and markers of various immune cell subsets

To further confirm the correlation among FARSB expression and different immune cells infiltration, we investigated the connection of FARSB expression and the levels with the expression of immune cell gene markers in HCC by using the TIMER database (Supplementary Table 1). These findings suggested that FARSB was highly associated with gene markers of most immune cells (Supplementary Figure 2A–2E). Moreover, we examined the link between FAESB and diverse T cell immune markers (Supplementary Table 2). The results suggest that FARSB expression was remarkably related to 29 of the 42 T cell markers in HCC. The data suggested that FARSB expression may influence the occurrence and development of hepatocellular carcinoma by regulating the degree of infiltration of T cells.

On the basis of previous reports, chemokines help to regulate immune cell migration and provide an anti-tumor immune response. Chemokines also help to generate and recruit immune cells, which forms a pro-tumor microenvironment. Thus, we assumed that T cells could be recruited by some specific cytokines, resulting in a pro-tumor microenvironment and a poor prognosis for HCC. Using the R software, we found that three chemokines and eight receptors were positively correlated with FARSB expression in HCC. Following that, based on plenty of assays, we summarized that CCL26, CX3CL1, and CCR8 can generate and recruit Th2. The scatter diagram showed concrete correlations (Supplementary Figure 2F). In addition, using immune-related chemokine and receptor expression in HCCLM3 cells, we discovered that CCL26, CX3CL1, and CCR8 expression were all down-regulated in shFARSB cells (Supplementary Figure 2G). That demonstrates CCL26, CX3CL1 and CCR8 expression level may be effected by FARSB expression. This suggested that FARSB may affect microenvironments primarily by influencing Th2 immune infiltration.

## FARSB PPI network (physical) and molecular docking demonstration

Generally speaking, protein interactions can perform biological functions and metabolic reactions. A FARSB protein interaction web had been established by GeneMANIA. (Supplementary Figure 3A) This result indicated FARSB had physical interaction with 11 different proteins. In these proteins, RPLP1, which has been validated that played an important role in HCC progression. In next step, cBioPortal database was utilized to research the secondary structure of FARSB and RPLP1 (Supplementary Figure 3B, 3C), which included various chemical modification sites, like phosphorylation, acetylation, ubiquitination, and methylation. Furthermore, we analyzed tertiary structure of protein FARSB and RPLP1 obtained from the PDB database, and the results of molecular docking showed that they can interact to form dimers (Supplementary Figure 3D).

## Correlation of FARSB expression with m6A related genes HNRNPC in HCC

Although HNRNPC is positively correlated with FARSB expression, differential expression of HNRNPC gene does not affect the survival of HCC patients, as shown in Supplementary Figure 4 (Figure 4), so we speculate that differential expression of HNRNPC gene has no significance in prognosis of patients.
