# Supplementary material for: FARSB serves as a novel hypomethylated and immune cell infiltration related prognostic biomarker in hepatocellular carcinoma
Source: Aging (Albany NY). 2023 Apr 3;15(8):2937–69. doi: 10.18632/aging.204619 (PMC10188347; doi:10.18632/aging.204619)
Supplement: Supplementary Figures [file aging-15-204619-s002.pdf]

SUPPLEMENTARY FIGURES

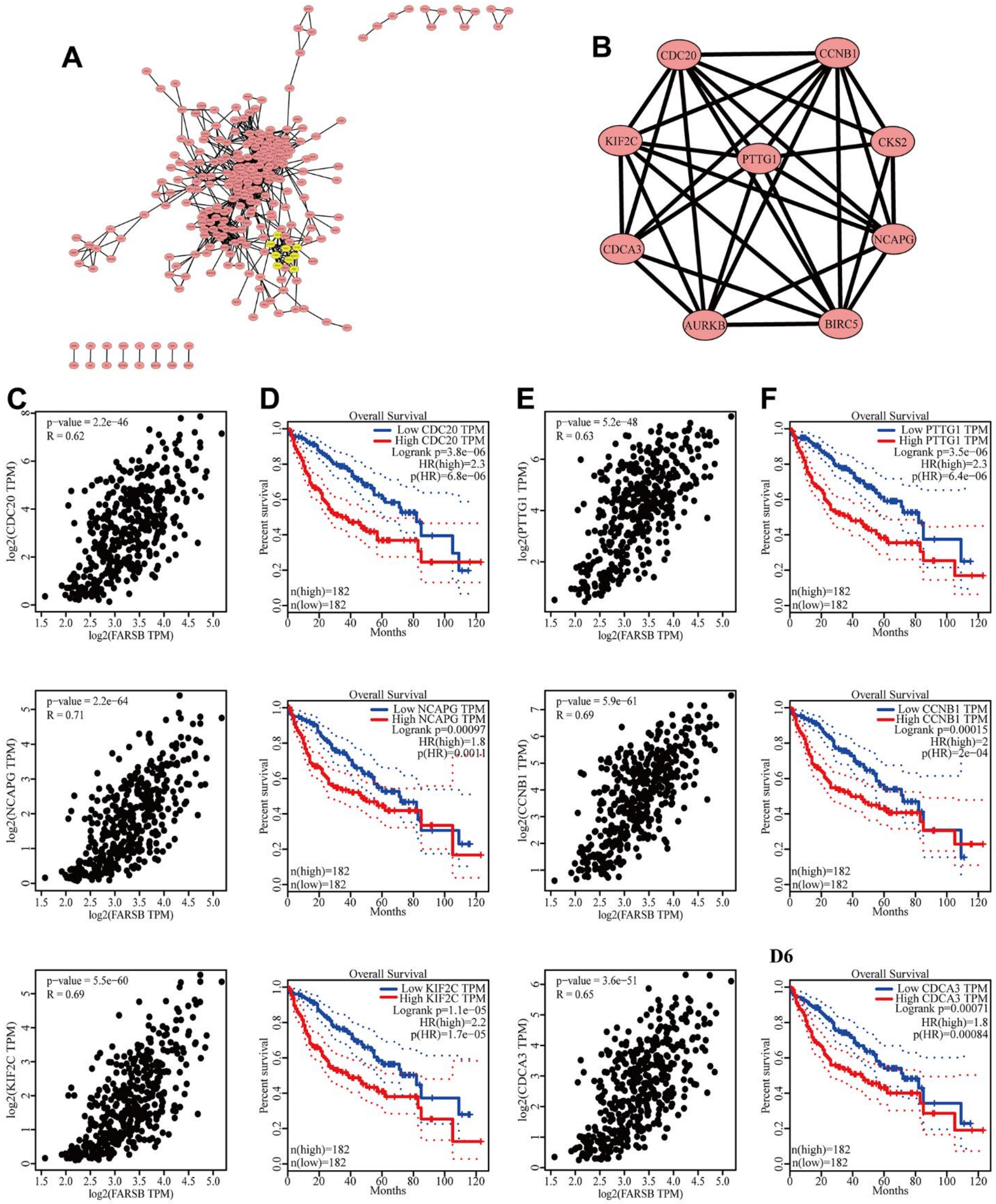

Supplementary Figure 1. Protein–protein interaction network of related genes (Top200) and analysis of hub genes in HCC. (**A**) Protein–protein interaction (PPI) network. (**B**) MCODE analysis. (**C1**-**C3**) Correlation between FARSB and the mRNA expression of CDC20, NCAPG, KIF2C in HCC determined using GEPIA. (**D**) Prognosis analysis of CDC20, NCAPG, KIF2C. (**E**) Correlation between FARSB and the mRNA expression of PTTG1, CCNB1, CDCA3 in HCC determined using GEPIA. (**F**) Prognosis analysis of PTTG1, CCNB1, CDCA3.

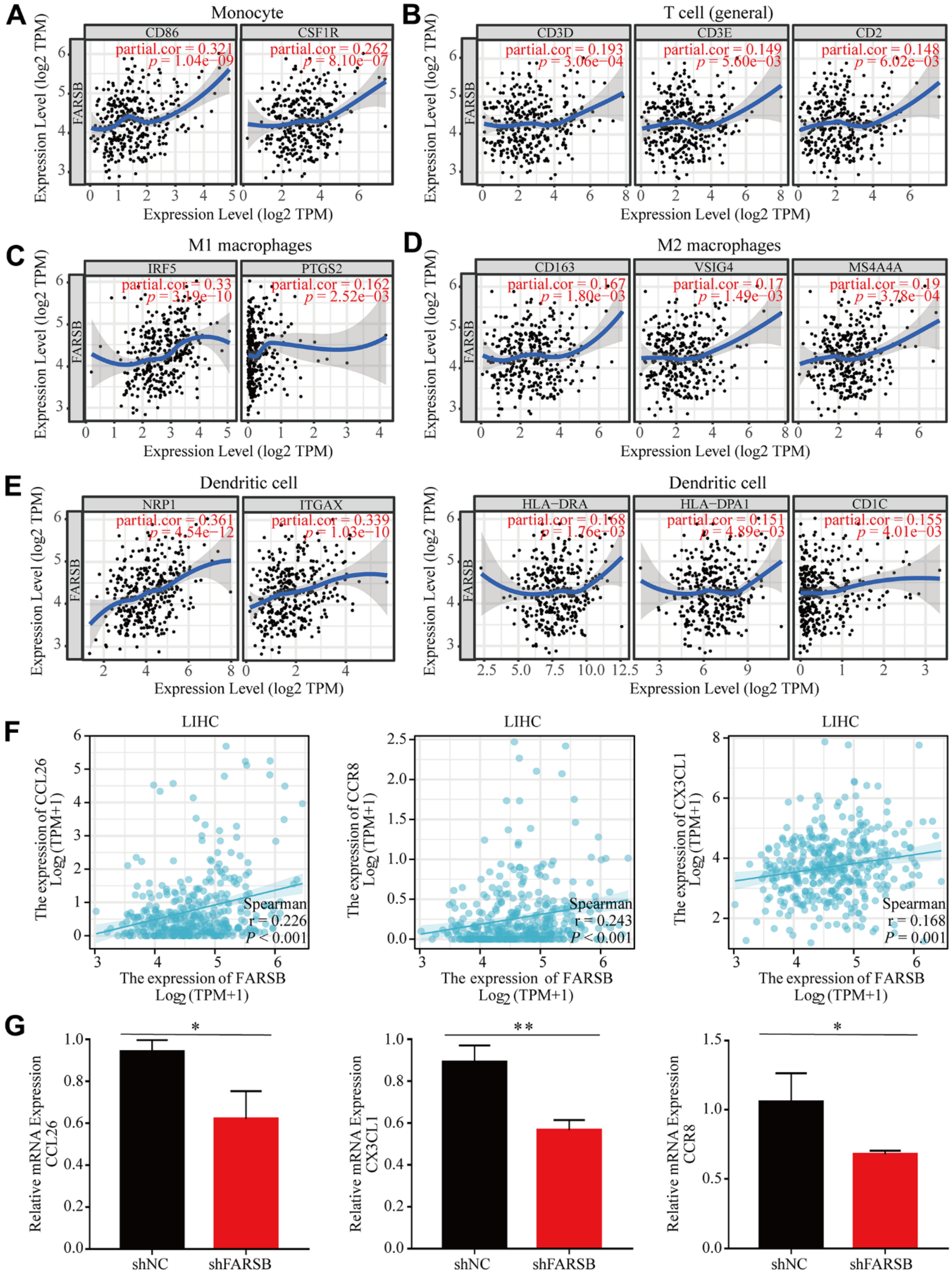

Supplementary Figure 2. Correlation of FARSB expression and the expression of marker genes of infiltrating immune cells. (**A**–**E**) The scatter plots show correlation between FARSB expression and the gene markers of (**A**) Monocytes (CD86 and CSF1R), (**B**) T cells (CD3D, CD3E, and CD2), (**C**) M1 macrophages (IRF5 and PTGS2), (**D**) M2 macrophages (CD163, VSIG4, and MS4A4A), (**E**) DCs (NRP1, ITGAX, HLA-DRA, HLA-DPA1, and CD1C) in HCC samples (P< 0.05). (**F**) Correlation between FARSB and CCL26, CX3CL1, CCR8 (**G**) CCL26, CX3CL1, and CCR8 expression in shFARSB HCCLM3 cells.

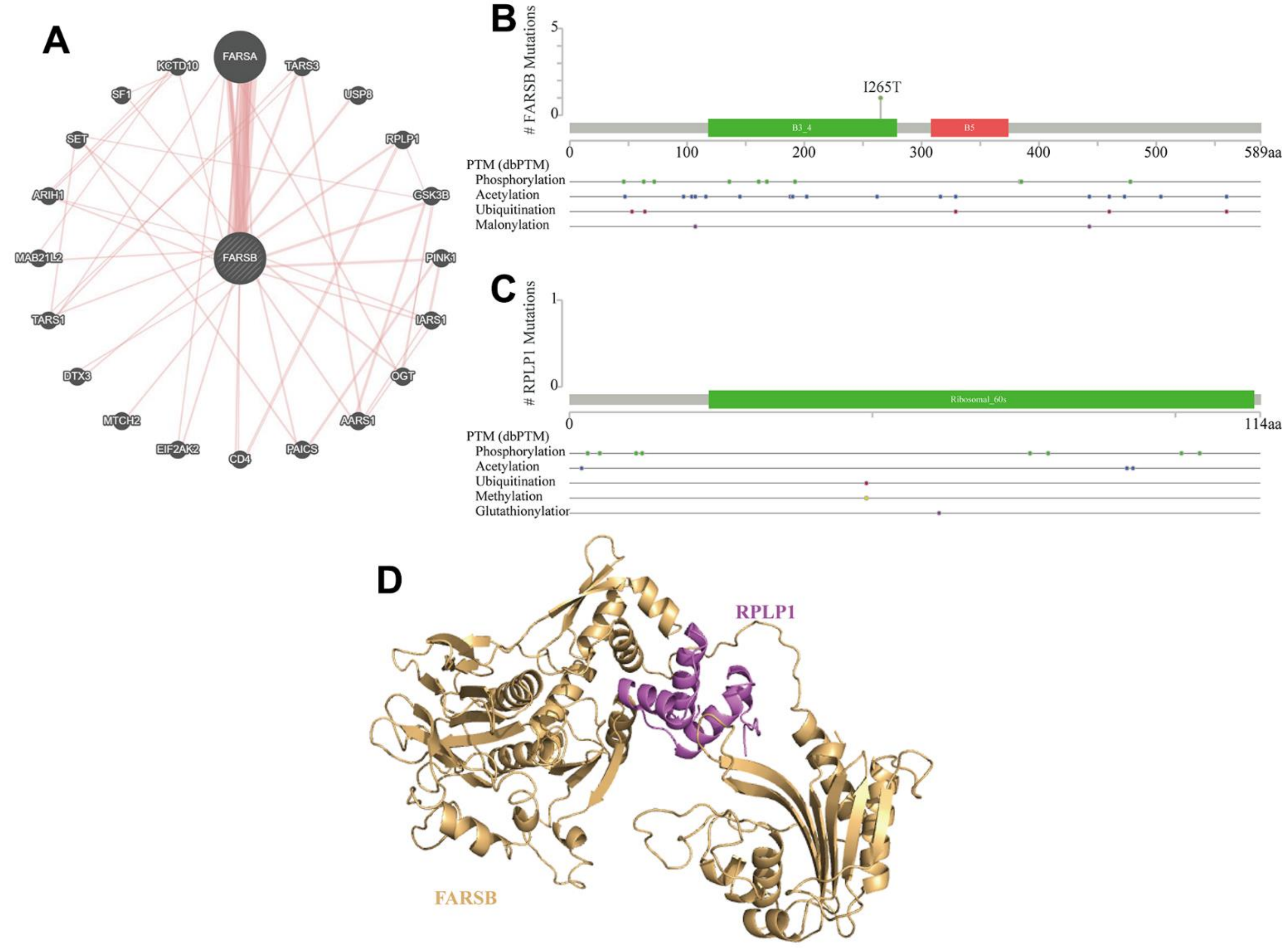

Supplementary Figure 3. Physical interaction of protein FARSB. (**A**) Gene interaction networks of FARSB. (**B**) Secondary protein structure of FARSB. (**C**) Secondary protein structure of RPLP1. (**D**) Tertiary structure of FARSB and RPLP1.

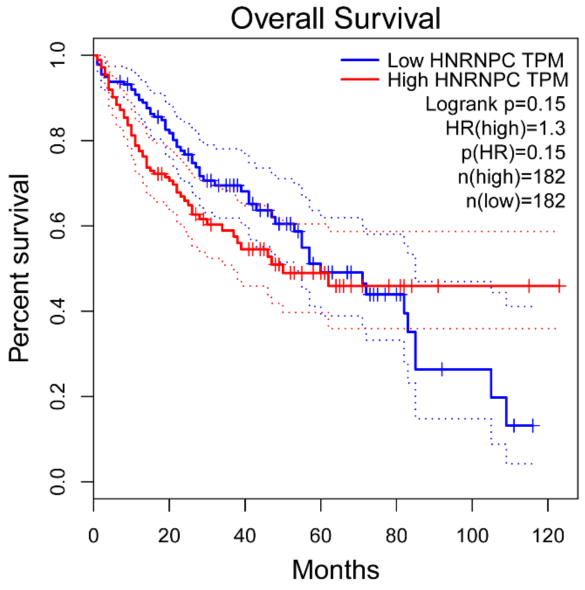

Supplementary Figure 4. Correlation of FARSB expression with HNRNPC. Kaplan-Meier curve of HNRNPC P = 0.15.
