# Supplementary material for: FARSB serves as a novel hypomethylated and immune cell infiltration related prognostic biomarker in hepatocellular carcinoma
Source: Aging (Albany NY). 2023 Apr 3;15(8):2937–69. doi: 10.18632/aging.204619 (PMC10188347; doi:10.18632/aging.204619)
Supplement: Supplementary Tables [file aging-15-204619-s003.pdf]

SUPPLEMENTARY TABLES

Supplementary Table 1. Correlation analysis between FARSB and gene markers of different types of immune cells in TIMER.

Dercriplion	Gene markers	LIHC			
		None		Purity	
		Cor	p	Cor	p
**B cell**	CD19	0.104214404	**0.044855959**	0.123222275	**0.022069116**
	CD79A	0.009098784	0.86134116	0.065729874	0.223313419
**T cell (general)**	CD3D	0.13202331	**0.010911543**	0.193218367	**0.000306289**
	CD3E	0.074806543	0.150425802	0.148842776	**0.005604865**
	CD2	0.07630841	0.14238052	0.14759635	**0.00602185**
**CD8+ T cell**	CD8A	0.054612411	0.294112414	0.10771003	**0.045588474**
	CD8B	0.051108027	0.326232917	0.103877546	0.053898025
**Monocyte**	CD86	0.227902052	**9.28E-06**	0.321041521	**1.04E-09**
	CSF1R	0.170413981	**0.000982587**	0.261877949	**8.10E-07**
**TAM**	CCL2	0.015387735	0.767683732	0.050524718	0.349455066
	CD68	0.198041898	**0.000123143**	0.260232284	**9.55E-07**
	IL10	0.19351693	**0.000176601**	0.263417657	**6.94E-07**
**M1**	IRF5	0.342494465	**1.20E-11**	0.330257517	**3.19E-10**
	PTGS2	0.089488535	0.085194697	0.162138779	**0.002522109**
**M2**	CD163	0.098560307	0.057878166	0.167432815	**0.00180423**
	VSIG4	0.103983914	**0.045334034**	0.170437314	**0.001485386**
	MS4A4A	0.107166481	**0.039098229**	0.190328312	**0.000378108**
**Neutrophils**	CEACAM8	0.092546723	0.075013287	0.105631942	**0.04994983**
	ITGAM	0.199818359	**0.000106654**	0.247485138	**3.28E-06**
	CCR7	-0.026760543	0.607393841	0.037815831	0.483865369
**Natural killer cell**	KIR2DL1	-0.061006804	0.24111643	-0.10800702	**0.044992072**
	KIR2DL3	0.05578367	0.283864589	0.077370512	0.151564189
	KIR2DL4	0.090469125	0.081815425	0.104520449	0.052422101
	KIR3DL1	0.01425062	0.784412039	0.02654771	0.623144373
	KIR3DL2	0.014435604	0.781683719	0.033815727	0.531315741
	KIR3DL3	-0.016457936	0.75203749	-0.025692059	0.63439114
**Dendritic cell**	HLA-DPB1	0.090507764	0.08168452	0.141637667	**0.008424901**
	HLA-DQB1	0.082969641	0.110607787	0.133072072	**0.013370518**
	HLA-DRA	0.106968143	**0.039464442**	0.167836695	**0.001758002**
	HLA-DPA1	0.090221537	0.082658262	0.151197369	**0.004887274**
	CD1C	0.107998249	**0.037593588**	0.154559938	**0.004005652**
	NRP1	0.341150496	**1.45E-11**	0.36117892	**4.54E-12**
	ITGAX	0.255023667	**6.42E-07**	0.338787898	**1.03E-10**

Supplementary Table 2. Correlation analysis between FARSB and gene markers of different types of T cells in TIMER.

Dercriplion	Gene markers	LIHC			
		None		Purity	
		Cor	p	Cor	p
**Th1**	TBX21	0.027399618	0.598838329	0.076901146	0.154069672
	STAT4	0.080902958	0.119804803	0.111209167	**0.038967497**
	STAT1	0.33446271	**3.80E-11**	0.367536869	**1.79E-12**
	TNF	0.203474714	**7.90E-05**	0.291471837	**3.50E-08**
	IFNG	0.109722286	**0.034630629**	0.157165131	**0.003424378**
**Th1-like**	HAVCR2	0.219261558	**2.04E-05**	0.317018555	**1.71E-09**
	IFNG	0.109722286	**0.034630629**	0.157165131	**0.003424378**
	CXCR3	0.086112846	0.09769529	0.132382119	**0.013862588**
	BHLHE40	0.084198559	0.105408017	0.091420974	0.089988453
	CD4	0.069225712	0.183360684	0.106725932	**0.047612294**
**Th2**	STAT6	0.238593057	**3.36E-06**	0.236723394	**8.82E-06**
	STAT5A	0.223716648	**1.36E-05**	0.248232305	**3.05E-06**
**Treg**	FOXP3	0.002835359	0.956593623	0.031934105	0.55442141
	CCR8	0.269570005	**1.34E-07**	0.331493275	**2.71E-10**
	TGFB1	0.227853526	**9.32E-06**	0.299185418	**1.45E-08**
**Tfh**	BCL6	0.288111274	**1.60E-08**	0.293088647	**2.92E-08**
	CXCR5	0.09887179	0.057087145	0.171443675	**0.001390734**
**Th17**	STAT3	0.119264959	**0.021581149**	0.145202592	**0.006901458**
	IL17A	0.039882134	0.443737788	0.036839617	0.495230296
**Resting Treg**	FOXP3	0.002835359	0.956593623	0.031934105	0.55442141
	IL2RA	0.194934962	**0.000157871**	0.286485447	**6.10E-08**
**Effector Treg T-cell**	FOXP3	0.002835359	0.956593623	0.031934105	0.55442141
	CCR8	0.269570005	**1.34E-07**	0.331493275	**2.71E-10**
	TNFRSF9	0.254961042	**6.46E-07**	0.319365353	**1.28E-09**
**Effector T-cell**	CX3CR1	0.184229114	**0.000360942**	0.210621573	**8.07E-05**
	FGFBP2	-0.042022031	0.419651109	-0.044003761	0.415208552
	FCGR3A	0.160675109	**0.001905542**	0.206903301	**0.000108354**
**Naïve T-cell**	CCR7	-0.026760543	0.607393841	0.037815831	0.483865369
	SELL	0.144842055	**0.005186843**	0.206638327	**0.000110629**
**Effector memory T-cell**	DUSP4	0.251501177	**9.25E-07**	0.331074964	**2.87E-10**
	GZMK	-0.038247325	0.462657278	0.011063177	0.837767547
	GZMA	-0.028474848	0.584568099	0.012693592	0.814265555
**Resident memory T-cell**	CD69	0.048514706	0.351411615	0.107365516	**0.046288607**
	CXCR6	0.048757811	0.34900051	0.114055716	**0.034198995**
	MYADM	0.381063361	**2.87E-14**	0.417125963	**5.90E-16**
**General**	CCR7	-0.026760543	0.607393841	0.037815831	0.483865369
**Memory T-cell**	SELL	0.144842055	**0.005186843**	0.206638327	**0.000110629**
	IL7R	0.057227702	0.2715653	0.122670126	**0.022676564**
**Exhausted T-cell**	HAVCR2	0.219261558	**2.04E-05**	0.317018555	**1.71E-09**
	LAG3	0.116249475	**0.025145259**	0.138354618	**0.010085575**
	CXCL13	0.035933406	0.490185659	0.076175696	0.158003611
	LAYN	0.168927859	**1.09E-03**	0.201609819	**1.63E-04**
